# Region-Specific Effects of Metformin on Gut Microbiome and Metabolome in High-Fat Diet-Induced Type 2 Diabetes Mouse Model

**DOI:** 10.3390/ijms25137250

**Published:** 2024-06-30

**Authors:** Meihui Cheng, Xianxian Jia, Lili Ren, Siqian Chen, Wei Wang, Jianwei Wang, Bin Cong

**Affiliations:** 1 Research Unit of Digestive Tract Microecosystem Pharmacology and Toxicology, National Institute of Pathogen Biology, Chinese Academy of Medical Sciences & Peking Union Medical College, Shijiazhuang 050017, China; 2 College of Forensic Medicine, Hebei Key Laboratory of Forensic Medicine, Collaborative Innovation Center of Forensic Medical Molecular Identification, Hebei Medical University, Shijiazhuang 050017, China; 3 National Health Commission Key Laboratory of Systems Biology of Pathogens and Christophe Mérieux Laboratory, National Institute of Pathogen Biology, Chinese Academy of Medical Sciences & Peking Union Medical College, Beijing 102629, China; 4 Department of Pathogen Biology, Institute of basic Medicine, Hebei Medical University, Shijiazhuang 050017, China

**Keywords:** metformin, type 2 diabetes mellitus (T2DM), gut microbiome, gut metabolome, microbiome-derived metabolites, gut regions

## Abstract

The glucose-lowering drug metformin alters the composition of the gut microbiome in patients with type 2 diabetes mellitus (T2DM) and other diseases. Nevertheless, most studies on the effects of this drug have relied on fecal samples, which provide limited insights into its local effects on different regions of the gut. Using a high-fat diet (HFD)-induced mouse model of T2DM, we characterize the spatial variability of the gut microbiome and associated metabolome in response to metformin treatment. Four parts of the gut as well as the feces were analyzed using full-length sequencing of 16S rRNA genes and targeted metabolomic analyses, thus providing insights into the composition of the microbiome and associated metabolome. We found significant differences in the gut microbiome and metabolome in each gut region, with the most pronounced effects on the microbiomes of the cecum, colon, and feces, with a significant increase in a variety of species belonging to *Akkermansiaceae*, *Lactobacillaceae*, *Tannerellaceae*, and *Erysipelotrichaceae*. Metabolomics analysis showed that metformin had the most pronounced effect on microbiome-derived metabolites in the cecum and colon, with several metabolites, such as carbohydrates, fatty acids, and benzenoids, having elevated levels in the colon; however, most of the metabolites were reduced in the cecum. Thus, a wide range of beneficial metabolites derived from the microbiome after metformin treatment were produced mainly in the colon. Our study highlights the importance of considering gut regions when understanding the effects of metformin on the gut microbiome and metabolome.

## 1. Introduction

Metformin is heralded as the foremost therapy for type 2 diabetes mellitus (T2DM) [1]. Increasingly, research indicates that the intestine plays a pivotal role in mediating metformin’s therapeutic effects [2,3,4]. Recent findings suggest that metformin profoundly modifies the gut microbiota’s composition and functionality [5,6,7]. These alterations in the microbiome are associated with changes in the intestinal metabolome, potentially influencing systemic metabolism and insulin responsiveness [8,9,10]. Nevertheless, the exact role of the gut microbiota and their metabolites in metformin’s mechanisms remains to be fully understood.

The gut microbiome is primarily composed of four phyla, Firmicutes, Bacteroidetes, Actinobacteria, and Proteobacteria, in both humans and mice [11,12]. The equilibrium among these microbial populations is essential for maintaining intestinal homeostasis and overall health [13]. Through their metabolic activities, these microbiota produce various metabolites, which significantly contribute to the host’s health [8,14,15]. Firmicutes mainly produce short-chain fatty acids, such as butyrate, propionate, and acetate, which help provide energy, maintain the intestinal barrier, and regulate the immune system [16,17]. Similarly, Bacteroidetes not only generate short-chain fatty acids but also produce immunomodulatory substances such as polysaccharides, which enhance gastrointestinal wellness [18,19]. Actinobacteria, especially *Bifidobacteria*, produce lactate and acetate to inhibit pathogen growth and support intestinal health [20,21]. Proteobacteria produce lipopolysaccharides, which can be pro-inflammatory in excess but contribute to immune regulation and gut function at normal levels [22,23].

Each section of the gastrointestinal tract has distinct physiological functions, and accordingly, its microbial and metabolite compositions are uniquely varied. These variations reflect changes in the gut microenvironment, influenced by factors like pH, oxygen concentration, available nutrients, intestinal motility, and immune responses [24,25]. The proximal small intestine is mainly responsible for the initial absorption of nutrients and has a relatively low microbial density [15,26]. The distal small intestine further absorbs nutrients and participates in the reabsorption of bile acids, displaying a more diverse microbial population [27]. The cecum, acting as a transition zone between the small and large intestines, exhibits a significant increase in microbial diversity and density, featuring a large presence of Firmicutes and Bacteroidetes, and starts fermenting indigestible substances, generating more short-chain fatty acids and other metabolites [27,28]. The colon, while reabsorbing water, also ferments indigestible dietary fibers through the activity of Firmicutes, Bacteroidetes, Actinobacteria, and Proteobacteria, generating various beneficial metabolites, which are crucial for maintaining gut health and modulating the host immune system [27,29].

The absorption rate of metformin varies across different regions of the gastrointestinal tract. It is predominantly absorbed in the proximal parts of the small intestine, especially in the duodenum, while its absorption in the distal small intestine and colon is comparatively lower [5,30]. Considering these differences in absorption rates across the intestinal regions, it is essential to undertake a comprehensive analysis of metformin’s impact on microbial community composition and the production of related metabolites in each intestinal region.

Previous research on the therapeutic effects of metformin was mainly focused on fecal samples, providing limited insight into the impact of metformin on the gut microbiota and related metabolites. Therefore, a more detailed examination of the complex interactions between metformin, the microbiota, and metabolites in various intestinal regions is necessary. Our study enhances this understanding by examining the impact of metformin on microbiota-derived metabolites in different intestinal regions as well as feces. Our approach offers significant advantages, utilizing high-precision sequencing of the full-length 16S rRNA gene for accurate microbial community identification and targeted metabolomics assays to precisely detect 415 key microbiome-derived metabolites in each intestinal region and in feces. This identification and analysis of the gut microbiota and associated metabolites in different intestinal regions and feces after metformin treatment lay the foundation for a thorough exploration of metformin’s effects.

## 2. Results

### 2.1. Microbial Composition of Metformin-Treated and Control Groups

Compared to that in the control group, weight gain was significantly slowed down in the high-fat diet-induced T2DM mouse model treated with metformin via gavage (Supplementary Figure S1A,B). In addition, fasting blood glucose, fasting insulin levels, and the insulin resistance index (HOMA-IR) were also improved in metformin-treated T2DM mice, as shown in Supplementary Figure S1C–E. These results suggest that metformin had a significant therapeutic effect on the high-fat diet-induced T2DM mouse model.

**Figure 1:**
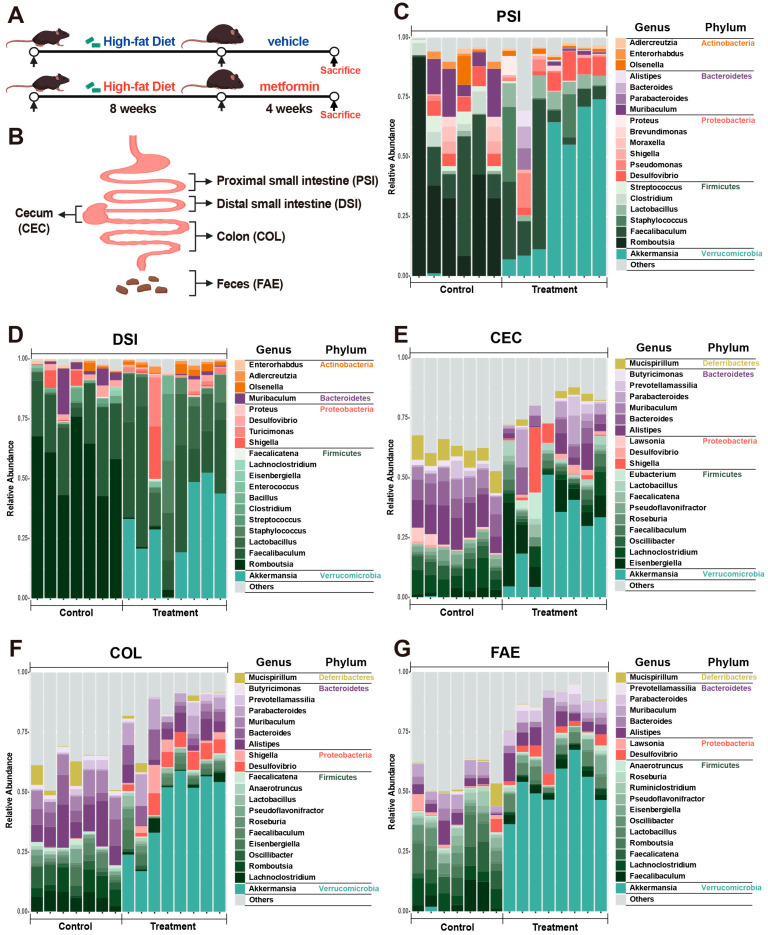
Comparison of the microbial composition in different intestinal regions and feces between metformin-treated and control groups. (**A**,**B**) Schematic of the experimental design. C57BL/6J mice were fed a high-fat diet for 8 weeks and then divided into two groups: one group received metformin via gavage, and the other received saline, serving as the treatment group (*n* = 7) and control group (*n* = 8), respectively, for 4 weeks. Samples from the proximal small intestine, distal small intestine, cecum, colon, and feces were collected at the end of the experiment for subsequent analysis. (**C**–**G**) The relative abundance of microbes in different intestinal parts and the feces of metformin-treated and control groups at the genus level—the top 20 genera are shown. PSI—proximal small intestine; DSI—distal small intestine; CEC—cecum; COL—colon; FAE—feces.

The microbial composition of the metformin-treated and control groups was analyzed by comprehensive sequencing of the full-length 16S rRNA gene from 75 microbial samples from various gastrointestinal regions in metformin-treated and control mice, including the proximal and distal small intestines, cecum, colon, and feces. Due to insufficient sequencing data for two samples, this study ultimately excluded these two samples and focused on detailed microbiome analyses of the remaining 73 samples. The scheme of the experimental procedure is shown in Figure 1A,B.

We identified a total of 786,902 high-quality reads across all 73 samples, which were clustered into 436 distinct operational taxonomic units (OTUs). Notably, in the proximal small intestine, 59 (26.2%) OTUs were common to both groups, while 87 (38.7%) and 79 (35.1%) OTUs were exclusive to the control and treatment groups, respectively (Supplementary Figure S2A); in the distal small intestine, there were 77 (33.6%) shared OTUs, with 90 (39.3%) and 62 (27.1%) unique OTUs for the control and treatment groups, respectively (Supplementary Figure S2B); in the cecum, 198 (55.9%) OTUs were shared, with 134 (37.9%) and 22 (6.2%) unique OTUs for the control and treatment groups, respectively (Supplementary Figure S2C); in the colon, there were 190 (40.7%) shared OTUs, with 143 (54.1%) and 18 (5.1%) unique OTUs for the control and treatment groups, respectively (Supplementary Figure S2D); and in the feces, 195 (54.3%) OTUs were shared, with 152 (42.3%) and 12 (3.3%) unique OTUs for the control and treatment groups, respectively (Supplementary Figure S2E).

This study further delved into the microbial composition at the phylum and genus levels, as demonstrated in Figure 1C–G. At the phylum level, Firmicutes and Verrucomicrobia were predominant in the small intestine of metformin-treated mice, contrasting with the control group’s dominance of Firmicutes. The cecum, colon, and feces of metformin-treated mice were characterized by the predominance of Bacteroidetes and Verrucomicrobia, whereas the control group showed a predominance of Bacteroidetes and Firmicutes. At the genus level, Faecalibaculum dominated in the proximal and distal small intestine of both treated and control mice, whereas Romboutsia was more abundant in the control group, and Akkermansia was more abundant in the metformin-treated group. Additionally, the control group exhibited a higher abundance of Alistipes and Mucispirillum in the cecum, Alistipes and Lachnoclostridium in the colon, and Faecalicatena and Romboutsia in feces. Post-metformin treatment, Akkermansia became the dominant genus in the cecum, colon, and feces.

**Figure 2:**
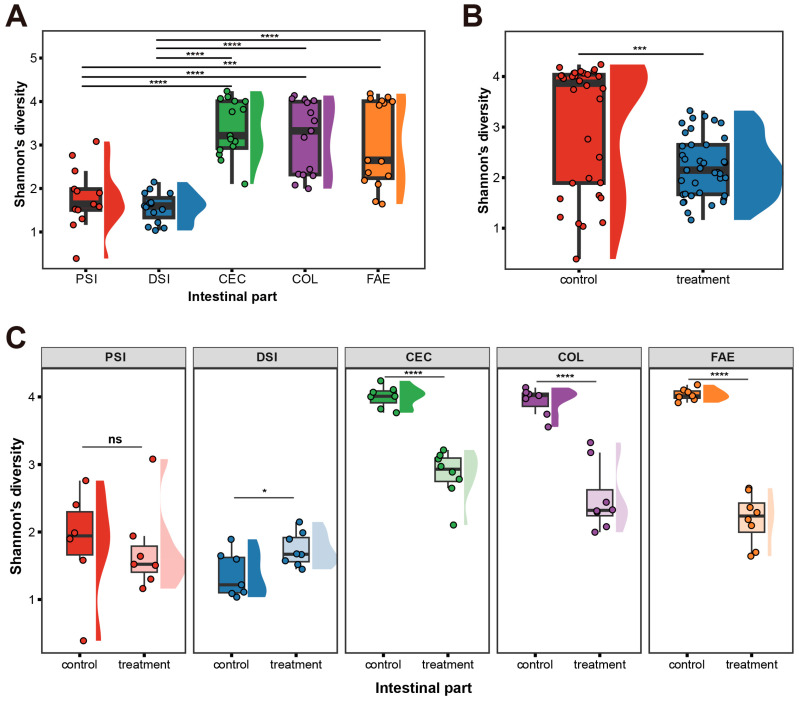
Alpha diversity analysis using the Shannon index. (**A**) Comparison of alpha diversity between different intestinal regions and feces. (**B**) Comparison between metformin-treated and control groups. (**C**) Comparison across various intestinal regions and feces after metformin treatment. PSI: proximal small intestine; DSI: distal small intestine; CEC: cecum; COL: colon; FAE: feces. Statistical differences were assessed using ANOVA for (**A**) and two-tailed unpaired *t*-tests for (**B**,**C**); significance levels are indicated as * *p* < 0.05, *** *p* < 0.001, **** *p* < 0.0001; ns means *p* > 0.05 (no significant difference).

Within-sample (α) phylogenetic diversity, as measured by the Shannon index, showed no significant differences between the proximal and distal small intestine, nor between the cecum, colon, and feces. However, there was a marked disparity in alpha diversity between the small intestine and other gastrointestinal sites, with lower diversity in the small intestine and significantly higher alpha diversity in the cecum, colon, and feces (Figure 2A). A notable difference in alpha diversity between the metformin-treated and control groups was also observed (Figure 2B). Further examination of the impact of metformin on alpha diversity across different intestinal sites revealed significant differences in the distal small intestine, cecum, colon, and feces following treatment, while the proximal small intestine remained unaffected (Figure 2C).

### 2.2. Alterations in Microbial Composition in Metformin-Treated Mice

**Figure 3:**
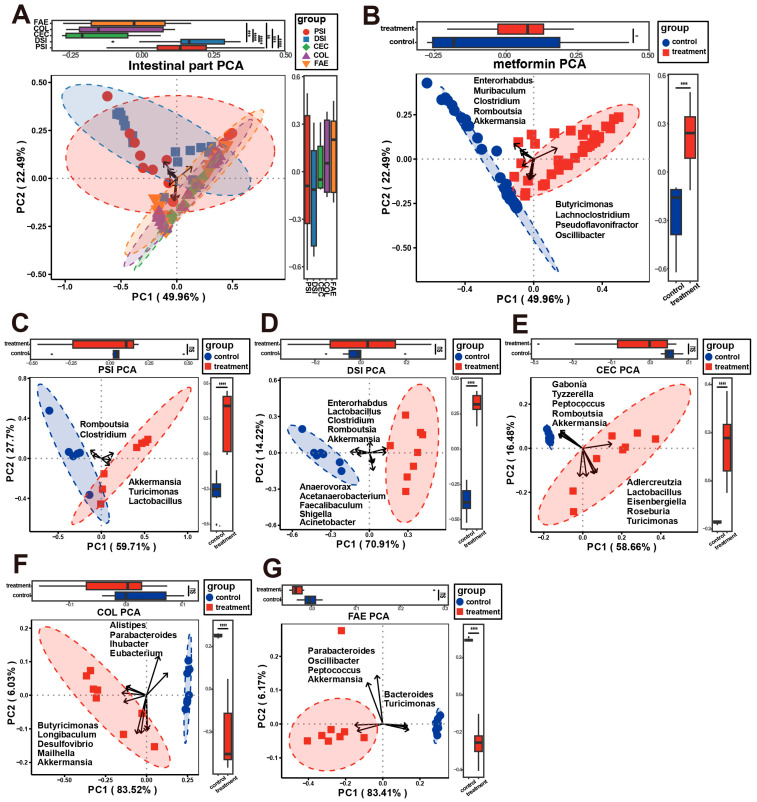
Beta diversity was estimated using principal components analysis. (**A**) Across different intestinal regions and feces. (**B**) Between metformin-treated and control groups. Beta diversity responses to metformin treatment at each site: (**C**) PSI—proximal small intestine; (**D**) DSI—distal small intestine; (**E**) CEC—cecum; (**F**) COL—colon; (**G**) FAE—feces. Statistical differences were evaluated using ANOVA for (**A**) and two-tailed unpaired *t*-tests for (**B**–**G**). Significance levels are indicated as * *p* < 0.05, ** *p* < 0.01, *** *p* < 0.001, **** *p* < 0.0001; ns means *p* > 0.05 (no significant difference).

Beta diversity was ascertained through ordination analysis. Principal component analysis (PCA) revealed distinct clustering of samples from the cecum, colon, and feces, which differed markedly from samples obtained from both regions of the small intestine (Figure 3A). Moreover, a significant divergence was observed between the metformin-treated and control groups, with Akkermansia, Romboutsia, Clostridium, Muribaculum, and Enterorhabdus emerging as the predominant identifiers in the metformin-treated group (Figure 3B). In the proximal small intestine, specifically after metformin treatment, Akkermansia, Turicimonas, and Lactobacillus were identified as the most prominent identifiers (Figure 3C). In the distal small intestine, the principal identifiers distinguishing the metformin-treated samples were Akkermansia, Romboutsia, Clostridium, Lactobacillus, and Enterorhabdus (Figure 3D). The cecum’s metformin-responsive identifiers included Akkermansia, Romboutsia, Peptococcus, Tyzzerella, and Gabonia (Figure 3E). In the colon, Akkermansia, Mailhella, Desulfovibrio, Longibaculum, and Butyricimonas were identified as key metformin-associated identifiers (Figure 3F). Similarly, in the feces, Akkermansia, Peptococcus, Oscillibacter, and Parabacteroides were the most significant identifiers post-metformin treatment (Figure 3G).

To delineate the disparate microbial compositions of the metformin-treated and control groups across four intestinal regions and the feces, we employed a detailed LEfSe analysis (LDA > 2 and *p*-value < 0.05), as shown in Supplementary Figure S3. Metformin had a stronger effect on the abundance of bacteria in the cecum, colon, and feces. In the proximal small intestine of the metformin-treated group, 25 bacterial species exhibited significant changes compared to the control group, with the majority belonging to Firmicutes (15/25, 60%) and Proteobacteria (5/25, 20%) at the phylum level, and the most affected families were Lachnospiraceae and Desulfovibrionaceae (Supplementary Figure S3A). In the distal small intestine, 35 bacterial species showed significant variations post-metformin treatment, predominantly within Firmicutes (19/35, 54.3%), Proteobacteria (6/35, 17.1%), and Bacteroidetes (5/35, 14.3%), with Lachnospiraceae, Ruminococcaceae, Lactobacillaceae, Desulfovibrionaceae, and Eggerthellaceae being the most affected families (Supplementary Figure S3B). In the cecum, 75 bacterial species were significantly altered, mostly within Firmicutes (47/75, 62.7%) and Bacteroidetes (13/75, 17.3%), with Lachnospiraceae, Ruminococcaceae, Rikenellaceae, and Clostridiaceae being the predominantly affected families (Supplementary Figure S3C). In the colon, 77 bacterial species were significantly changed following metformin treatment, with the largest changes observed in Firmicutes (49/77, 63.6%) and Bacteroidetes (15/77, 19.5%) and the most impacted families being Lachnospiraceae, Ruminococcaceae, Erysipelotrichaceae, Desulfovibrionaceae, and Rikenellaceae (Supplementary Figure S3D). In the feces, 84 species exhibited significant alterations, primarily in Firmicutes (61/84, 72.6%) and Bacteroidetes (10/84, 11.9%), with Lachnospiraceae, Ruminococcaceae, and Clostridiaceae being the most altered families (Supplementary Figure S3E).

**Figure 4:**
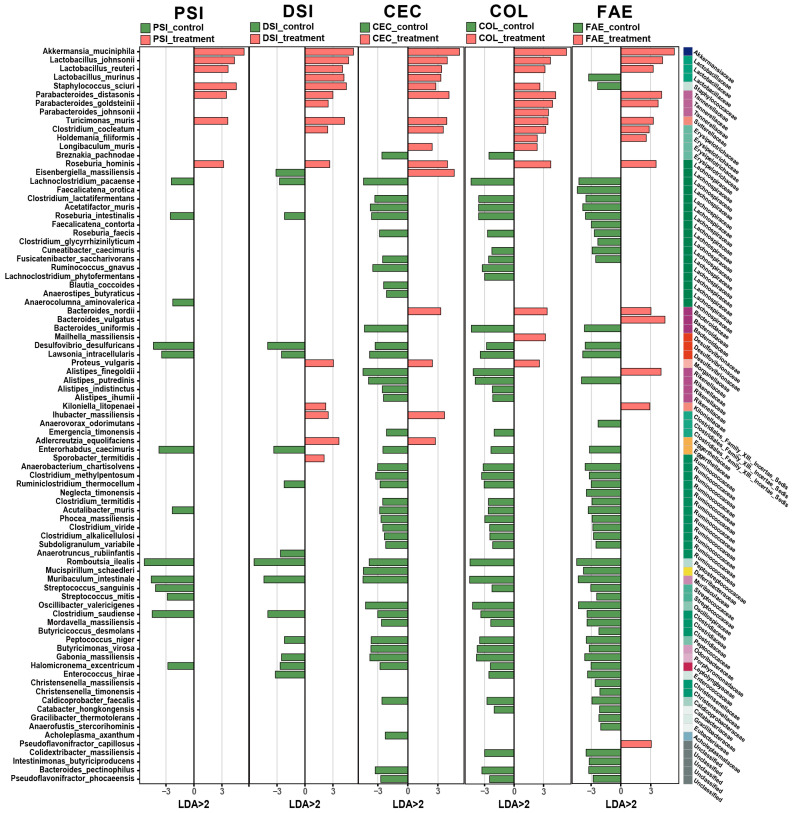
Summary of differentially abundant bacterial species in response to metformin treatment in different intestinal regions and feces. Only the bacterial species with an absolute LDA >2 in at least one of the comparison sets were included. Red bars indicate bacterial species that increased in abundance in metformin-treated mice, while green bars represent species that decreased in abundance. PSI—proximal small intestine; DSI—distal small intestine; CEC—cecum; COL—colon; FAE—feces.

Further analysis of the expression changes in the gut microbiota at the species level revealed that, following metformin treatment, there were a total of 85 bacterial species exhibiting significant differences across various intestinal segments and feces (LDA > 2 and *p*-value < 0.05), as shown in Figure 4. The variation in bacterial species was less pronounced in both parts of the small intestine compared to the cecum, colon, and feces. Following metformin treatment, there was a predominant increase in the expression of bacterial strains from the Akkermansiaceae, Lactobacillaceae, Tannerellaceae, and Erysipelotrichaceae families. Conversely, strains exhibiting a decline in expression were largely associated with the Lachnospiraceae and Ruminococcaceae families. Of these, Akkermansia muciniphila, Lactobacillus johnsonii, Lactobacillus reuteri, Parabacteroides distasonis, Turicimonas muris, and Roseburia hominis exhibited significantly higher expression in all intestinal regions and feces post-metformin treatment. Additionally, Staphylococcus sciuri expression increased in all intestinal regions but decreased in the feces after metformin administration. Post-metformin treatment, there was a notable decline in the expression of most bacterial strains in the cecum, colon, and feces, with strains Clostridium lactatifermentans, Acetatifactor muris, Bacteroides uniformis, Alistipes putredinis, Oscillibacter valericigenes, and Butyricimonas virosa experiencing the most substantial changes, whereas in the small intestine, expression levels predominantly stayed constant.

### 2.3. Alterations in Metabolomic Composition in Metformin-Treated Mice

**Figure 5:**
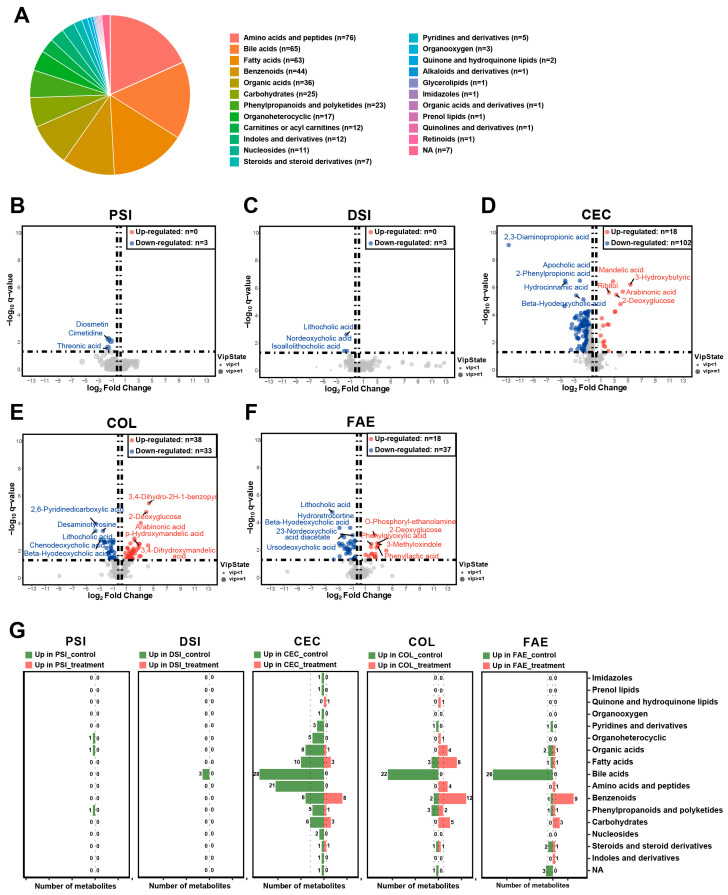
Comparative metabolomics analysis illustrating changes in gut metabolites between metformin-treated and control groups. (**A**) Categories of identified gut metabolites and their counts. Volcano plots display the number of deregulated metabolites in the (**B**) proximal small intestine, (**C**) distal small intestine, (**D**) cecum, (**E**) colon, and (**F**) feces, comparing the metformin-treated group with the control group. (**G**) Butterfly diagram showing differences in various classes of metabolites. Differential metabolites were obtained by screening with (1) VIP ≥ 1 in the OPLS-DA model; (2) fold change ≥ 1.2 or ≤0.83; and (3) q-value < 0.05. PSI—proximal small intestine; DSI—distal small intestine; CEC—cecum; COL—colon; FAE—feces.

We quantified 415 metabolites that covered a wide range of significant metabolites in the intestine using targeted metabolomic techniques, encompassing 22 distinct classes of metabolites (Supplementary Table S1). The most abundant categories were amino acids and peptides (76 metabolites), followed closely by bile acids and fatty acids (65 and 63 metabolites respectively), as shown in Figure 5A. 

Utilizing orthogonal partial least squares discriminant analysis (OPLS-DA), a method renowned for enhancing group differentiation [31], we discerned notable disparities in metabolite profiles between metformin-treated and control groups. This was evident in various intestinal regions and the feces, as shown in Supplementary Figure S4A, with no overfitting observed (Supplementary Figure S4B), thus validating the model’s precision.

The comparative analysis uncovered pronounced metabolomic alterations across various intestinal regions and the feces following metformin treatment (Supplementary Figure S5). Screening criteria for differential metabolites were VIP ≥ 1, fold change ≥ 1.2 or ≤0.83, and q-value < 0.05. In both regions of small intestine, there were hardly any changes in metabolites. However, in the cecum, colon, and feces, a substantial number of metabolites exhibited significant alterations, with the highest quantity of metabolic changes occurring in the cecum and colon (Figure 5B–F). In the proximal small intestine, notable decreases were observed in diosmetin, cimetidine, and threonic acid levels post-metformin treatment, with no significant increases reported (Figure 5B). Similarly, in the distal small intestine, lithocholic acid, nordeoxycholic acid, and isoallolithocholic acid levels significantly decreased, with no notable increases (Figure 5C). However, in the cecum, the number of differential metabolites was significantly higher, with 120 metabolites altered significantly post-treatment, including 18 metabolites, like mandelic acid, 3-hydroxybutyric acid, and arabinonic acid, exhibiting pronounced increases and 102 metabolites showing significant decreases (Figure 5D). The colon revealed alterations in 71 metabolites, with 38, like 3,4-dihydro-2H-1-benzopyran-2-one and 2-deoxyglucose, significantly increasing and 33 significantly decreasing (Figure 5E). Fecal analysis showed 55 metabolites with significant changes, 18 of which, such as O-phosphoryl-ethanolamine, increased significantly, while 37, including lithocholic acid and hydroretrocortine, decreased significantly (Figure 5F).

By categorizing these differential metabolites, we observed a predominant decrease in bile-acid-class metabolites, particularly in the cecum, colon, and feces, post-metformin treatment. In contrast, benzenoid-class metabolites were significantly increased in the cecum, colon, and feces. Additionally, it is noteworthy that the highest number of significantly reduced metabolites was seen in the cecum, while the colon had the highest number of significantly increased metabolites. Specifically, in the cecum, metabolites from classes like amino acids and peptides, carbohydrates, benzenoids, fatty acids, organic acids, and organoheterocyclics primarily decreased, whereas, in the colon, metabolites from amino acids and peptides, fatty acids, carbohydrates, and organic acids predominantly increased. Furthermore, distinct from other areas, in the colon post-metformin treatment, there was a noticeable increase in most fatty-acid-class metabolites (Figure 5G).

**Figure 6:**
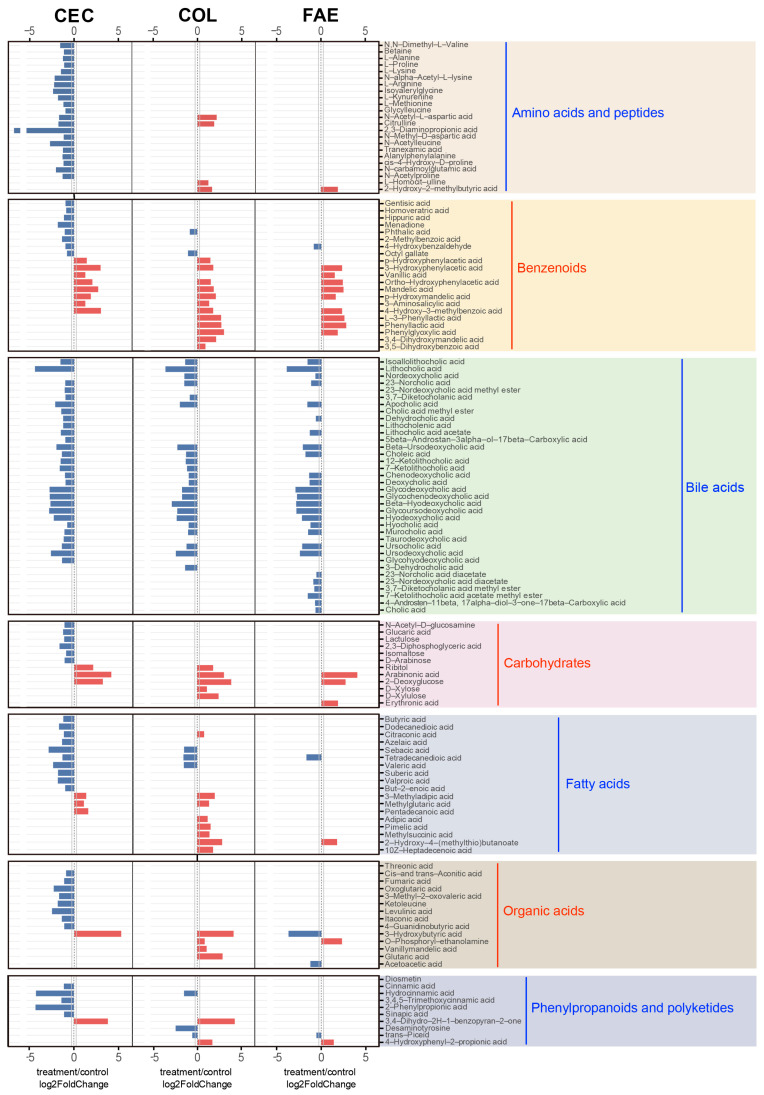
Summary of differentially abundant gut microbiota-related metabolites across the cecum, colon, and feces in response to metformin treatment. Differential metabolites were obtained by screening with (1) VIP ≥ 1 in the OPLS-DA model; (2) fold change ≥ 1.2 or ≤ 0.83; and (3) q-value < 0.05. Red bars represent increased metabolites in metformin-treated mice, and blue bars represent decreased metabolites. CEC—cecum; COL—colon; FAE—feces.

After metformin treatment, the main categories of differential metabolites and the expression differences in all differential metabolites within these categories were detailed in the cecum, colon, and feces (Figure 6). Notably, bile acid metabolites, particularly lithocholic acid, exhibited significant alterations in the cecum, colon, and feces post-treatment. After metformin treatment, in the cecum, colon, and feces, most metabolites belonging to benzenoids, carbohydrates, and fatty acids significantly increased, with the most notable changes observed in the colon. For example, in the colon, the most pronounced increases were seen in benzenoid-class metabolites such as L-3-phenyllactic acid, phenyllactic acid, and phenylglyoxylic acid; in carbohydrates, arabinonic acid and 2-deoxyglucose showed the most significant upward trends; and among fatty-acid-class metabolites, 2-hydroxy-4-(methylthio)butanoate and 3-methyladipic acid exhibited the most marked increases. These data suggest that although metformin had the broadest impact on the metabolites in the cecum, primarily exerting an inhibitory effect, its stimulatory effects on metabolites were mainly manifested in the colon.

### 2.4. Correlation Analysis of Microbiome and Metabolome Data

**Figure 7:**
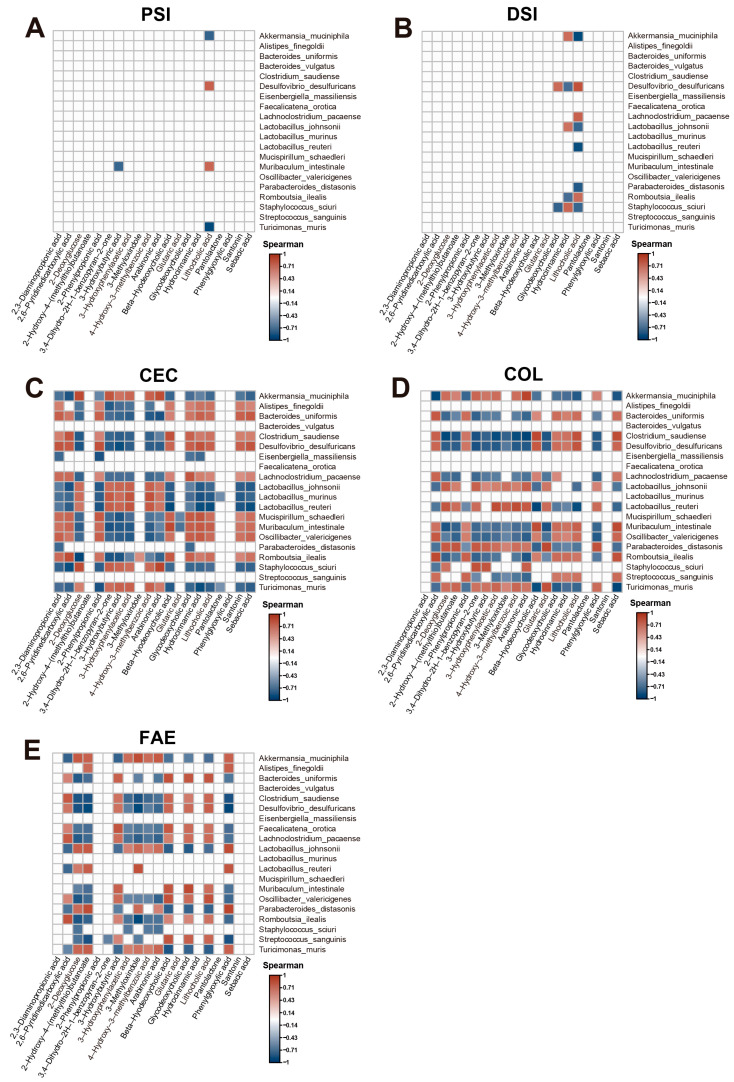
Correlation between differential microbiota and differential metabolites across different intestinal regions and the feces following metformin treatment. Correlation heatmap showing the interactions between the top 20 differential metabolites and the top 20 differential microbiota in (**A**) the proximal small intestine, (**B**) the distal small intestine, (**C**) the cecum, (**D**) the colon, and (**E**) the feces. Only correlations with a q-value less than 0.05 are displayed. The intensity of the color indicates Spearman’s correlations (positive: red; negative: blue). PSI—proximal small intestine; DSI—distal small intestine; CEC—cecum; COL—colon; FAE—feces.

Using correlation heatmaps, this study delves into the relationships between the top 20 most significantly altered microbiota and metabolites following metformin treatment, as shown in Figure 7.

We observed notable variations in the correlations between altered microbiota and metabolites after metformin treatment across different regions of the intestine, including the proximal small intestine, distal small intestine, cecum, colon, and feces. In our previous study, through microbiome analysis, we found that the abundance of Akkermansia was significantly higher in all intestinal regions and fecal samples after treatment with metformin, and Akkermansia was identified as the predominant identifier in all intestinal regions and fecal samples (Figure 1 and Figure 3). Furthermore, several studies have highlighted that Akkermansia muciniphila plays an important role in treatment with metformin [32,33,34,35]. In the proximal small intestine, no metabolites exhibited positive interactions with Akkermansia muciniphila (Figure 7A). In the distal small intestine, the metabolite positively correlated with Akkermansia muciniphila was hydrocinnamic acid (Figure 7B). Compared to those in the small intestine, the interactions between the microbiota and their metabolites in the cecum, colon, and feces were more complex following metformin treatment. 

In the cecum, the types of metabolites associated with Akkermansia muciniphila changed, showing positive correlations with metabolites such as 2-deoxyglucose, 3,4-dihydro-2H-1-benzopyran-2-one, 3-hydroxybutyric acid, 3-hydroxyphenylacetic acid, 4-hydroxy-3-methylbenzoic acid, and arabinonic acid (Figure 7C). However, compared to that in the cecum, in the colon following metformin treatment, the number of metabolites associated with Akkermansia muciniphila significantly increased, showing positive correlations with metabolites including 2-deoxyglucose, 2-hydroxy-4-(methylthio)butanoate, 3,4-dihydro-2H-1-benzopyran-2-one, 3-hydroxybutyric acid, 3-hydroxyphenylacetic acid, 4-hydroxy-3-methylbenzoic acid, arabinonic acid, and phenylglyoxylic acid (Figure 7D). In the feces after metformin treatment, the number of metabolites associated with Akkermansia muciniphila decreased, and the correlated metabolites shifted to 2-deoxyglucose, 2-hydroxy-4-(methylthio)butanoate, 3-hydroxyphenylacetic acid, 3-methyloxindole, 4-hydroxy-3-methylbenzoic acid, arabinonic acid, and phenylglyoxylic acid (Figure 7E). These data indicate significant differences in the types of metabolites associated with Akkermansia muciniphila across different intestinal regions after metformin treatment, with the colon having the highest diversity of positively correlated metabolites.

## 3. Discussion

**Figure 8:**
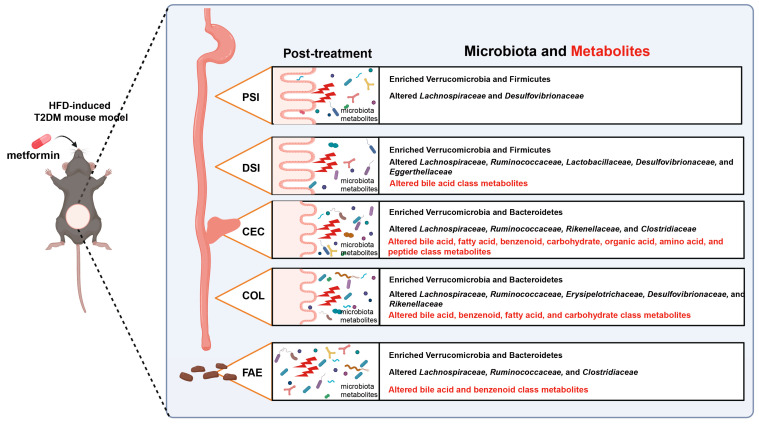
Schematic diagram summarizing the findings of this study. Treatment with metformin in mice with HFD-induced T2DM results in significant alterations in microbiota and metabolites across various gastrointestinal regions, including the proximal small intestine, distal small intestine, cecum, colon, and feces. Abbreviations: HFD, high-fat diet; T2DM, type 2 diabetes mellitus; PSI, proximal small intestine; DSI, distal small intestine; CEC, cecum; COL, colon; FAE, feces. Image created with BioRender.com, with permission.

There is growing evidence that metformin acts predominantly in the gut and that metformin treatment leads to significant changes in gut microbiota and metabolite composition [6,7,8,9,36]. However, detailed assessments of metformin’s effects on the microbiome and metabolome throughout various segments of the gastrointestinal tract are still lacking. Our research highlights extensive alterations in the configurations of microbes and metabolites across different intestinal regions and the feces following metformin treatment in mice with T2DM induced by a high-fat diet. These alterations include changes in the composition and diversity of the microbiota, the abundance of specific taxa, and the metabolomic profiles at various intestinal locations. We have summarized the key findings of this study in a schematic diagram, as shown in Figure 8.

The alpha diversity analyses showed that the diversities of microbial populations within the proximal and distal small intestines were low and not significantly different from each other. However, the microbial populations in the cecum, colon, and feces exhibited greater diversity compared to those in the small intestine, aligning with prior research [37]. Furthermore, metformin’s influence on the diversity of microbial communities in the cecum, colon, and feces surpassed its impact on the microbial diversity in both parts of the small intestine. Beta diversity analyses reinforced these findings, illustrating significant variances in microbial communities between metformin-treated and control groups across various gut regions and feces. These differences in microbial composition and diversity highlight metformin’s region-specific effects on modulating the gut microbiota.

Treatment with metformin in mice with T2DM led to significant changes in microbial populations across different regions of the intestines and in the feces. Bacteria such as Akkermansia, Romboutsia, and Clostridium emerged as main identifiers in the metformin-treated mice, signifying metformin’s selective effect on certain microbiota. The Akkermansia genus is well known for its beneficial role in metabolic health [38]. Importantly, our research found Akkermansia to be one of the key markers for metformin treatment across all intestinal regions and fecal samples. This finding is consistent with existing studies that highlight its role in improving glucose homeostasis and reducing inflammation [32,35,39].

After treatment with metformin, significant changes in the abundance of bacterial species were observed across different intestinal regions and in the feces. While previous studies have indicated metformin’s primary absorption in the small intestine [5], our findings reveal a more pronounced shift in microbial composition, especially in the cecum, colon, and feces after metformin treatment, surpassing the alterations seen in the small intestine. This variation might be due to the physiological environment of the small intestine, which has a lower microbial density and conditions that favor aerobic and microaerobic organisms [40,41]. Thus, the physiological conditions of different intestinal segments may influence the varying modulatory effects of metformin on the gut microbiota.

In various gut regions, the primary bacterial phyla that showed significant changes in species abundance after metformin treatment were Firmicutes and Bacteroidetes. An imbalance in the Firmicutes-to-Bacteroidetes ratio is believed to be linked with obesity and a range of metabolic disorders [42]. Specifically, at the family level, metformin treatment led to noticeable shifts in the populations of Lachnospiraceae and Ruminococcaceae. The Lachnospiraceae family, in particular, has been associated with T2DM in both human and animal studies [43]. The experimental introduction of a Lachnospiraceae strain into germ-free mice caused significant increases in fasting blood glucose levels and liver and mesenteric fat mass, accompanied by decreases in plasma insulin levels and homeostatic model assessment of beta-cell function (HOMA-β) scores [44]. Thus, the therapeutic efficacy of metformin may also be attributed to its regulatory effect on Lachnospiraceae populations. Full sequencing of the V1–V9 region of the 16S rRNA gene in the microbiota allows for precise species-level identification, refining the accuracy of studies assessing metformin’s influence on the gut microbiome [45]. Significantly, post-metformin treatment, there was a marked increase in the presence of species such as Akkermansia muciniphila, Lactobacillus johnsonii, Lactobacillus reuteri, Parabacteroides distasonis, Turicimonas muris, and Roseburia hominis across all intestinal regions and fecal samples. This finding corroborates the notion that metformin enhances the proliferation of Akkermansia muciniphila in the gut, a species famed for its mucin-degrading capabilities and subsequent fermentation into acetate and propionate, affecting vital host processes like immune modulation and lipid metabolism [33,34,35,38]. The hypoglycemic effect of metformin is possibly linked to the enriched presence of Akkermansia muciniphila [35]. Furthermore, Parabacteroides distasonis has been shown to significantly reduce symptoms related to obesity, insulin resistance, lipid metabolism disorders, and nonalcoholic fatty liver disease (NAFLD) in obesity model organisms [46]. Our research underscores that metformin elevates the levels of Parabacteroides distasonis throughout the gut. Moreover, Lactobacillus johnsonii and Lactobacillus reuteri, known for their gut-barrier-strengthening and anti-inflammatory properties [47,48,49,50], suggest a potential pathway through which metformin exerts its therapeutic effects.

Using targeted metabolomics, we quantified metformin’s influence on gut microbiota-related metabolites across intestinal segments and feces. Metformin’s metabolic changes were minimal in the small intestine due to low microbial activity [41]. In contrast, the cecum displayed a notable increase in differential metabolites, correlating with higher microbial densities [25]. The most pronounced changes were in the cecum and colon, where there was a greater increase in metabolites in the colon compared to the cecum, reflecting metformin’s impact on nutrient processing and microbial composition [7,24,27]. Notably, the bile acid metabolite lithocholic acid, associated with insulin resistance [51], was significantly reduced in the intestinal segments and feces after metformin treatment. This reduction may illuminate the underlying mechanism of metformin’s blood-glucose-lowering effect.

Following metformin therapy, a marked increase in various metabolites, including benzenoids, carbohydrates, and fatty acids, was noted predominantly in the cecum, colon, and feces, with the colon exhibiting the most significant changes. The cecum serves as the initial segment of the large intestine, participating in the early stages of fermentation. In contrast, the colon represents the primary site for intense and dynamic microbial activity within the gut, where these microbes generate a wide array of metabolites through the fermentation of dietary fibers and other food components that the small intestine does not absorb [24,25,27]. This intensive microbial activity likely accounts for the higher quantity of upregulated metabolites found in the colon. Our study revealed that in the colon treated with metformin, there was a substantial increase in various metabolites related to the gut microbiota, including amino acids and peptides, fatty acids, carbohydrates, benzenoids, and organic acids. However, the exact roles of these metabolites during metformin treatment remain to be thoroughly investigated.

Correlation analysis between the microbiome and metabolome has unveiled the intricate interactions within the gastrointestinal tract after metformin treatment. A synergistic relationship is evident between the intestinal microbes and metabolites, with the complexity of these interactions being notably greater in the cecum, colon, and feces than in both parts of the small intestine. It has been shown that Akkermansia muciniphila is associated with the action of metformin [35]. In the colon, we found that multiple metabolites were positively correlated with Akkermansia muciniphila. Notably, 2-deoxyglucose showed a positive correlation with Akkermansia muciniphila across the cecum, colon, and feces. Animal experiments and early clinical trials suggest that 2-deoxyglucose may improve symptoms of diabetes and related metabolic indicators to some extent [52,53,54]. Besides 2-deoxyglucose, several other metabolites also demonstrate positive interactions with Akkermansia muciniphila, including 3,4-dihydro-2H-1-benzopyran-2-one, 3-hydroxybutyric acid, 3-hydroxyphenylacetic acid, 4-hydroxy-3-methylbenzoic acid, arabinonic acid, and phenylglyoxylic acid. While there is no direct evidence currently linking these metabolites to metformin, they may play significant roles in various metabolic pathways. For instance, 3-hydroxybutyric acid is crucial in ketone metabolism, and metformin might exert its effects through the regulation of fatty-acid metabolism, thereby potentially influencing ketone body production [55,56,57]. Our research detailed the alterations in the microbiota and metabolome within various intestinal segments and the feces following metformin administration, providing important references for subsequent research on metformin’s gastrointestinal effects. 

However, our study did not examine the long-term effects of metformin on the gut microbiome and metabolome, which are critical for understanding its potential in chronic disease management and require further exploration and research in the future. Our study emphasizes the importance of examining the effects on specific gut regions, which are extremely relevant to human medicine. Understanding how metformin alters the microbiome and metabolome in these specific areas can help researchers design better therapeutic strategies, potentially including drug delivery systems targeted to specific gut regions in patients with T2DM.

## 4. Materials and Methods

### 4.1. Animal Experiments

C57BL/6J mice exhibit a high susceptibility to high-fat diets, leading to the development of obesity and type 2 diabetes mellitus (T2DM) in experimental models. Therefore, C57BL/6J mice were selected for this study. C57BL/6J mice obtained from Beijing Vital River Laboratory Animal Technology Co. were kept in a controlled environment with SPF conditions, including a temperature of 21 ± 2 °C and humidity between 40% and 70%. A 12-h light cycle (8 am to 8 pm) was implemented. The mice were accommodated in individually ventilated cages (Fengshi, China), housing 3–4 mice each. They were given an unrestricted amount of a 60% high-fat diet (D12492, Research Diets), as well as free access to water.

T2DM was induced by maintaining the mice on a 60% high-fat diet for 8 weeks. Targeted metabolomics analyses require a minimum of 6 replicates. Therefore, subsequently, we randomly divided the mice into two groups: one group of mice was gavaged with metformin (D150959, Sigma, Castleford, UK) at a dose of 300 mg/kg/day (*n* = 8), and the other group was gavaged with an equal amount of sterile water daily as a control (*n* = 7). This program was continued for 4 weeks along with the high-fat diet. The experimental design is illustrated in Figure 1A. The body weights of the mice were recorded every two days during metformin treatment. At the end of metformin treatment, all experimental mice were fasted for 12–16 h, followed by the measurement of fasting blood glucose levels in venous blood (Roche) and the detection of fasting insulin levels in the serum using an ELISA kit (90080, Crystal Chem, Grove Village, IL, USA). The insulin resistance index (HOMA-IR) was calculated using the following formula: HOMA-IR = fasting blood glucose (mmol/L) × fasting insulin level (mIU/L)/22.5.

### 4.2. Sample Collection

For all experimental mice, including high-fat-diet-induced T2DM mice treated with metformin (*n* = 8) and gavage vehicle-treated control mice (*n* = 7), the contents of their proximal small intestine, distal small intestine, colon, cecum, and feces were collected. Therefore, eight and seven biological replicates of the experimental and control groups per intestinal segment were used for full-length sequencing analysis of 16S rRNA genes and targeted metabolomics analysis, respectively. The detailed sample collection protocol is described below: feces were gathered from all mice before euthanasia and stored in sterile tubes at −80 °C. The mice were then euthanized under CO_2_ anesthesia, and their intestinal regions (proximal small intestine, distal small intestine, cecum, and colon) were isolated using sterile scissors. The contents of each segment were extracted with sterile forceps and directly transferred into sterile tubes. These samples were immediately frozen at −80 °C for subsequent analysis. A schematic of the sample collection sites is depicted in Figure 1B.

### 4.3. DNA Isolation and Full-Length 16S rRNA Gene Amplification

DNA extraction from the collected samples was performed using the QIAamp Fast DNA Stool Mini Kit (51604, Qiagen, Venlo, The Netherlands), adhering to the manufacturer’s protocol. DNA concentrations were measured with an enzyme laboratory apparatus using the DNA BR kit (Q33230, Invitrogen, Carlsbad, CA, USA). For each sample, 10 ng of genomic DNA was subjected to PCR amplification of the full-length 16S rRNA gene using the universal primers 27F (5′-AGRGTTYGATYMTGGGCTCAG-3′) and 1492R (5′-RGYTACCTTGTTACGACTT-3′). The PCR products underwent 1% agarose gel electrophoresis, followed by purification using magnetic beads. Subsequently, the purified samples were sequenced utilizing the Pacbio platform.

### 4.4. Data Analysis of Full-Length 16S rRNA Gene Sequences

To obtain clean data, the raw sequencing data underwent processing. Initially, cutadapt software (v2.6) was used to intercept primer and junction contamination in the reads that aligned with the primers, resulting in the extraction of fragments within the target region. Next, filtering was performed using readfq (v1.0) [58]. A method was employed to lower the quality of reads by analyzing windows of 30 bp each. If the average quality value within a window fell below 20, the end sequences of the reads were trimmed from the start of the window. Reads with a final length less of than 75% of the original length were then discarded. Furthermore, reads containing N and low-complexity reads were removed, and the resulting clean data underwent quality control using iTools Fqtools fqcheck (v0.25). The software FLASH (v1.2.11) was utilized to merge read pairs from double-end sequencing into a single sequence by identifying overlaps and to extract tags from the high-variance region. The splicing parameters included a minimum matching length of 15 bp and an allowable mismatch rate of 0.1 for the overlap region [59]. Following this, the combined tags were grouped into operational taxonomic units (OTUs) through the utilization of the program USEARCH (v7.0.1090) [60]. This process entails clustering with UPARSE at a 97% similarity threshold to acquire the characteristic sequences of OTUs and subsequently eliminating chimeric sequences produced during PCR amplification with the assistance of UCHIME (v4.2.40). OTU representative sequences were compared to all tags using the usearch_global method to calculate the abundance statistics of OTUs in each sample. The representative OTU sequences were then annotated against the NCBI database (20170709) using BLCA software (v2.1) [61], with a confidence threshold of 0.6 to refine the annotation results. The refined OTUs were subsequently utilized for further analyses. 

Venn diagrams of OTUs were plotted using the VennDiagram package in R (v4.2.2) software. Shannon indices for alpha diversity were calculated using Mothur (v1.31.2)’s summary.single command [62], and then the results were imported into R (v4.2.2) software and the ggplot2 package was used to read and visualize these alpha diversity indices. Principal component analysis (PCA) and visualization were performed using the FactoMineR package in R (v4.2.2) software. In a Linux setting, LEfSe was utilized to identify the significant bacterial taxa that distinguished the metformin-treated group from the control group based on criteria of LDA > 2 and a *p*-value less than 0.05. Strains with significant differences and grouping names were imported into R (v4.2.2) software and visualized using the ggplot2 package.

### 4.5. Targeted Metabolomics Profiling

A comprehensive quantitative analysis was conducted on 415 metabolites that covered a wide range of significant metabolites in the intestine (Supplementary Table S1). The initial phase involved the meticulous preparation of intestinal and fecal samples through a process of homogenization, dissociation, and centrifugation. The subsequent UPLC-MS analyses employed a Waters ACQUITY UPLC I-Class Plus system (Waters, Milford, MA, USA) in conjunction with a high-precision QTRAP6500 Plus mass spectrometer (SCIEX, Framingham, MA, USA). The chosen column for this procedure was the BEH C18 (2.1 mm × 10 cm, 1.7 μm, Waters), utilizing a mobile phase comprising 0.1% formic acid in water (Liquid A) and a 30% isopropanol–acetonitrile mixture (Liquid B). The QTRAP 6500 Plus instrument was operated in both positive and negative ion-switching modes, effectively identifying metabolites as they were eluted from the column. Detailed analyses of chromatographic peak areas and retention times were conducted using Skyline (v.21.1.0.146) software [63]. The derived results underwent further refinement using metaX software (v3.2), facilitating the extraction of pertinent compounds and quantitative data suitable for rigorous analysis. The metabolite identification results were informatively annotated using the HMDB database. During the mass spectrometry uptake of the samples, a certain number of quality control samples was added for data quality assessment. OPLS-DA analysis was conducted on metabolite data with the ropls package in R (v4.2.2) software to obtain VIP values. The fold change and q-value of metabolites in the treatment group were determined relative to those the control group, primarily utilizing the Benjamini–Hochberg (BH) approach. Differential metabolite screening was conducted using the VIP value from the OPLS-DA model and the fold change and q-value in univariate analysis. The criteria for screening were VIP ≥ 1 in the OPLS-DA model, fold change ≥ 1.2 or ≤0.83, and q-value < 0.05. The results of the differential metabolites were visualized using the ggplot2 package in R (v4.2.2) software.

### 4.6. Correlation Analysis

Spearman’s rank correlation analysis was utilized to investigate the interactions between the top 20 differential metabolites and the top 20 differential microbiota across various intestinal regions (proximal small intestine, distal small intestine, cecum, colon, and feces) in both metformin-treated and control groups. After adjusting for multiple testing using the false discovery rate (FDR) correction, the analysis selectively showed only correlations with q-values less than 0.05. The correlation results were visualized using the corrplot package in R (v4.2.2) software.

## 5. Conclusions

In conclusion, our study describes the spatial differences in the impact of metformin on the gut microbiome and metabolome in mice with high-fat-diet-induced T2DM. The most pronounced effects on the microbiome were observed in the cecum, colon, and feces after metformin treatment, with significant increases in the abundance of several species of Akkermansiaceae, Lactobacillaceae, Tannerellaceae, and Erysipelotrichaceae. In addition, the cecum, colon, and feces showed the highest abundance of the phyla Verrucomicrobia and Bacteroidetes after metformin treatment. Metabolomics analysis showed that metformin had the most pronounced effect on metabolites in the cecum and colon, with several beneficial metabolites belonging to carbohydrates, fatty acids, and benzenes having elevated levels in the colon; however, levels of most of the metabolites were reduced in the cecum. Thus, a wide range of beneficial metabolites derived from the microbiome after metformin treatment are produced mainly in the colon. Our study further emphasizes the importance of considering the spatial differences in the gastrointestinal tract when researching the impact of metformin on the gut microbiome and microbiome-derived metabolites.

## Data Availability

The data that support the findings of this study are deposited in the Bioproject (National Center for Biotechnology Information (NCBI)) at https://submit.ncbi.nlm.nih.gov/subs/sra/SUB14377791/overview (accessed on 1 June 2024), reference number [PRJNA1100315].

## Supplementary Materials

The following supporting information can be downloaded at: https://www.mdpi.com/article/10.3390/ijms25137250/s1.
