# Supplementary material for: Region-Specific Effects of Metformin on Gut Microbiome and Metabolome in High-Fat Diet-Induced Type 2 Diabetes Mouse Model
Source: Int J Mol Sci. 2024 Jun 30;25(13):7250. doi: 10.3390/ijms25137250 (PMC11241422; doi:10.3390/ijms25137250)
Supplement: Supplementary file 1 [file ijms-25-07250-s001.zip › Supplementary Figures.pdf]

Supplementary Figures

Supplementary Figure S1

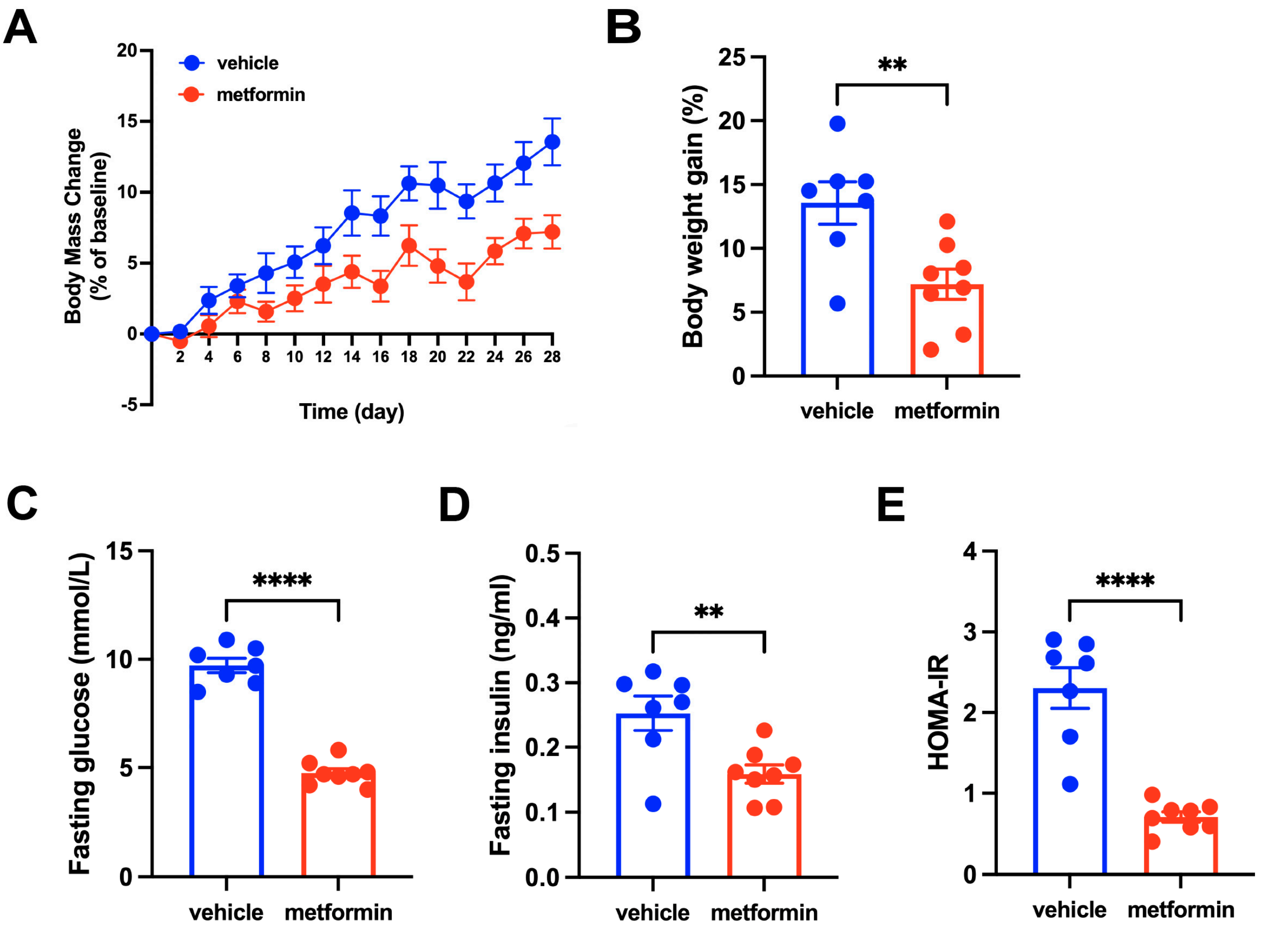

Supplementary Figure S1. Metformin significantly inhibits obesity and glucose disorders induced by a high-fat diet. After 4 weeks (28 days) of treatment, the effects on high-fat diet-induced T2DM mouse models treated with vehicle (n=7) and metformin (n=8) are shown in body mass change (A), rate of weight gain (B), fasting blood glucose levels (C), fasting insulin levels (D) and insulin resistance index (HOMA-IR) (E). All data are represented as mean ± SEM. Statistical differences were assessed using two-tailed unpaired *t*-tests, with significance levels indicated as $^{**}P < 0.01$, $^{****}P < 0.0001$.

Supplementary Figure S2

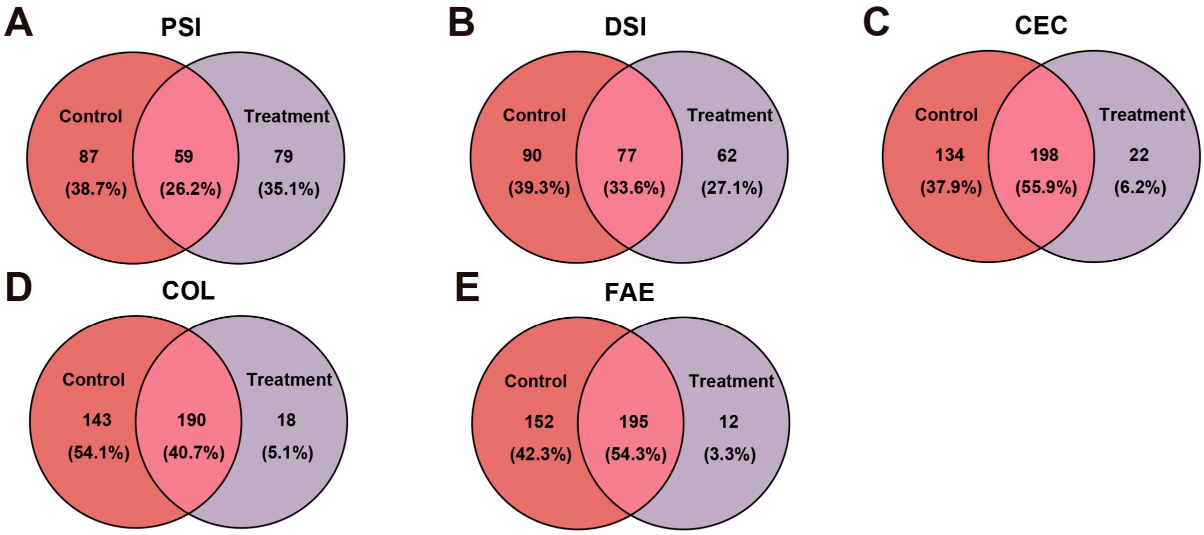

Supplementary Figure S2. Venn diagrams show the richness of OTUs and the overlap in microbial communities between the control and treatment groups: (A) PSI, proximal small intestine; (B) DSI, distal small intestine; (C) CEC, cecum; (D) COL, colon; (E) FAE, feces.

Supplementary Figure S3

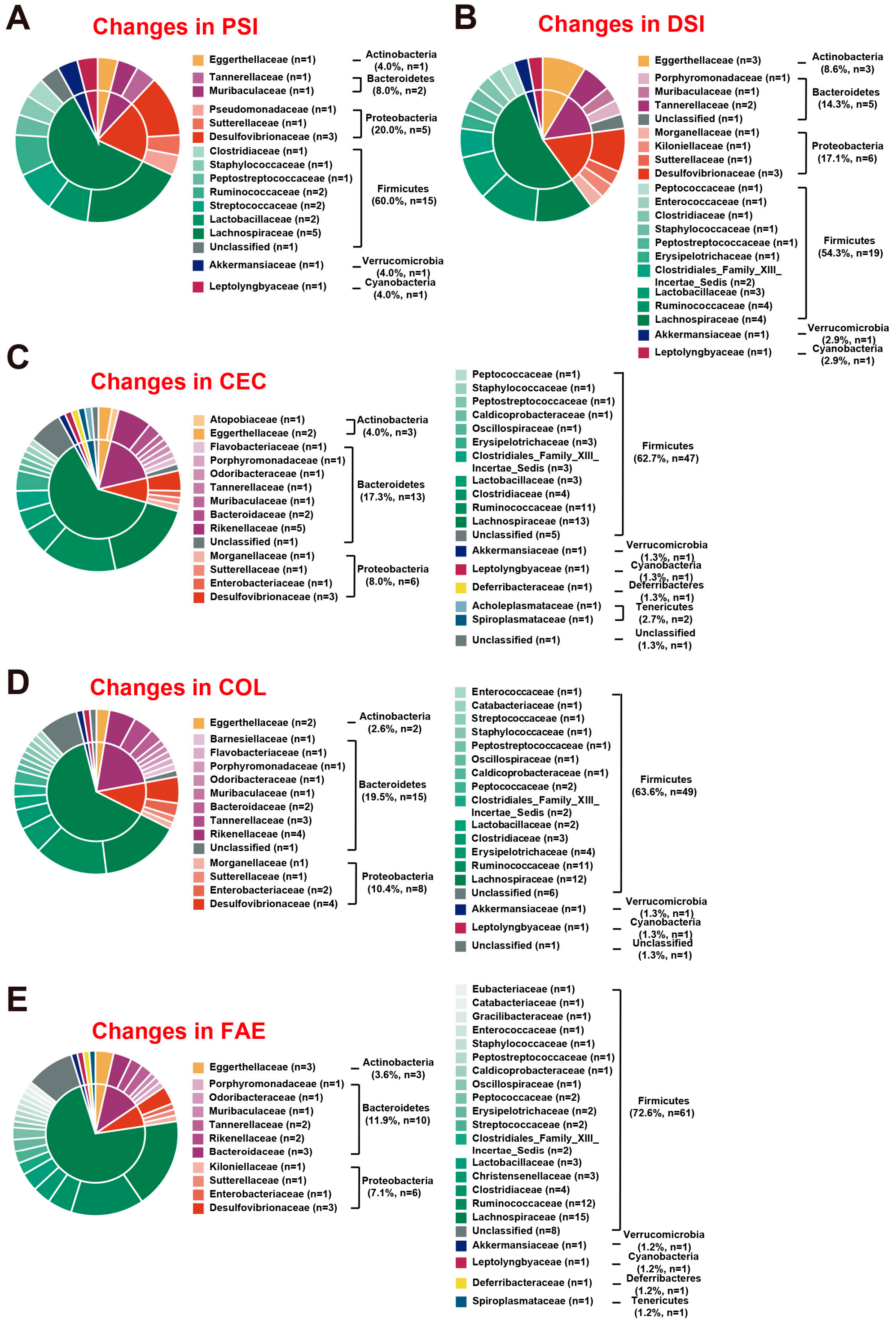

Supplementary Figure S3. Pie charts depicting changes in bacterial species composition in the metformin-treated group at the phylum and family levels. The charts illustrate the alterations in bacterial distribution within the metformin-treated group across different regions of the gastrointestinal tract: (A) PSI, proximal small intestine; (B) DSI, distal small intestine; (C) CEC, cecum; (D) COL, colon; (E) FAE, feces.

Supplementary Figure S4

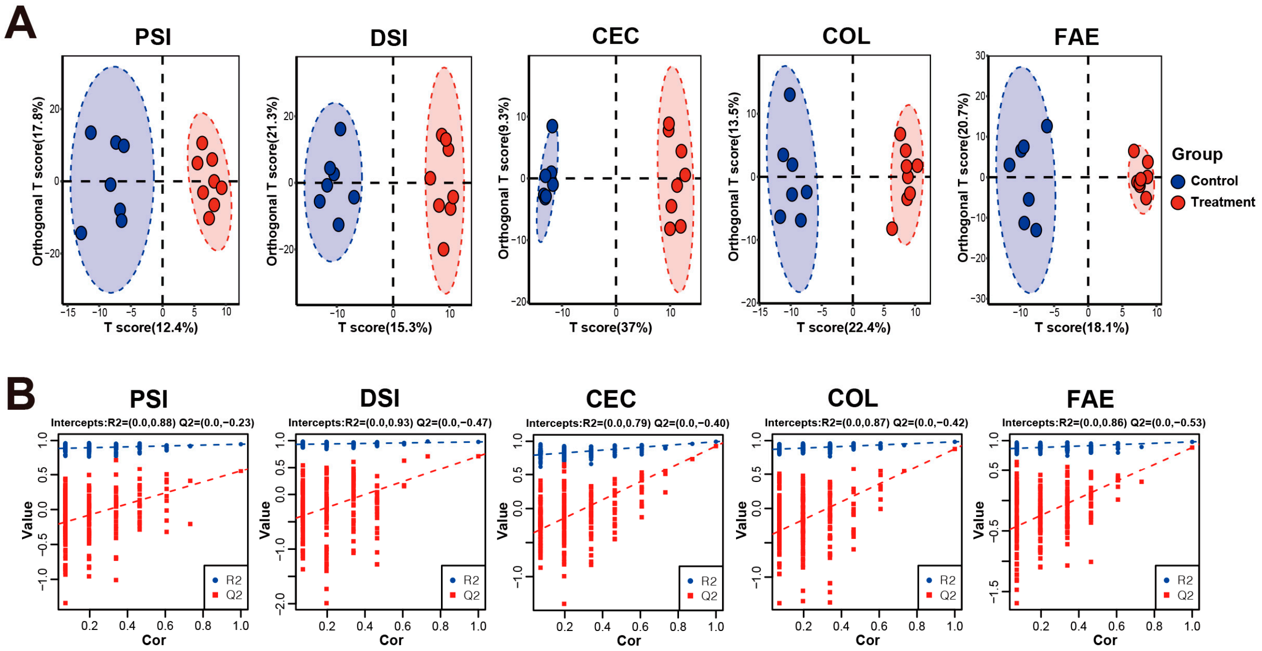

Supplementary Figure S4. OPLS-DA analysis comparing metformin-treated and control groups. (A) OPLS-DA scores of plots of metabolites in different intestinal regions and faeces from the metformin-treated and control groups. (B) Validation of OPLS-DA model by 200 response permutation testing. The x-axis means the correlation coefficient between the original y variable and the permutated y variable, and the y-axis is the value of R2 and Q2. PSI-Proximal small intestine; DSI-Distal small intestine; CEC-Cecum; COL-Colon; FAE-Feces.

Supplementary Figure S5

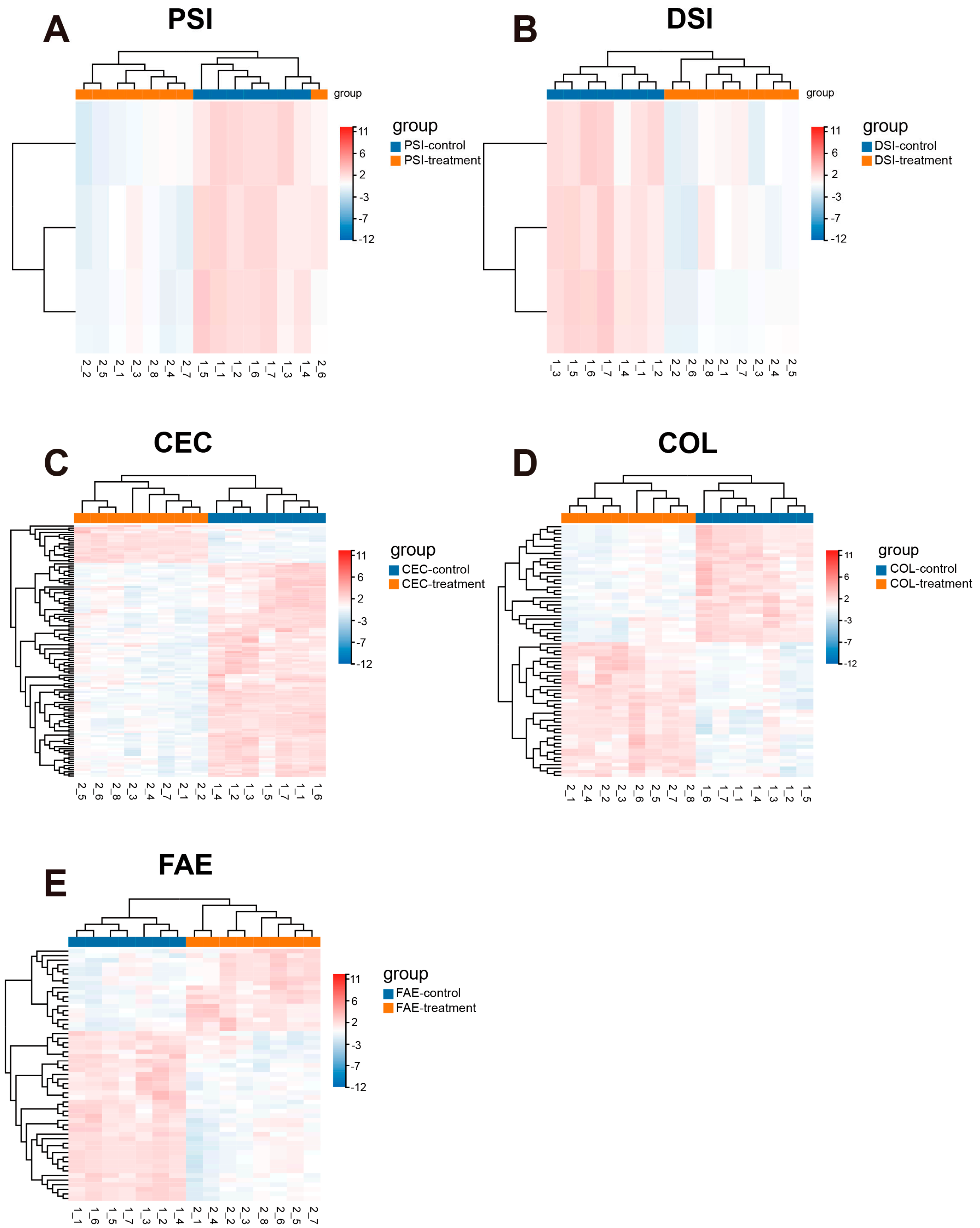

Supplementary Figure S5. Heatmap analysis of metabolite differences in metformin-treated and control groups. This heatmap illustrates the variations in metabolite profiles between the metformin-treated and control groups across various intestinal regions: (A) PSI, proximal small intestine; (B) DSI, distal small intestine; (C) CEC, cecum; (D) COL, colon; (E) FAE, feces.
