# Supplementary material for: Region-Specific Effects of Metformin on Gut Microbiome and Metabolome in High-Fat Diet-Induced Type 2 Diabetes Mouse Model
Source: Int J Mol Sci. 2024 Jun 30;25(13):7250. doi: 10.3390/ijms25137250 (PMC11241422; doi:10.3390/ijms25137250)
Supplement: Supplementary file 1 [file ijms-25-07250-s001.zip › Supplementary Table S1.pdf]

Supplementary Table S1. Details of 415 metabolites for targeted metabolomics analysis.

Name	CAS number	Class	HMDB
Glycodeoxycholic acid	360-65-6	Bile acids	HMDB0000631/C1087-000
Phosphoenolpyruvic acid	820-11-1	Carbohydrates	HMDB0000807
Tartaric acid	87-69-4	Carbohydrates	HMDB0000956
3-Dehydrocholic acid	2304-89-4	Bile acids	HMDB0000502/C1272-000/CAS2304-89-4
Chenodeoxycholic acid	474-25-9	Bile acids	HMDB0000518/C0940-000/CAS474-25-9
Taurodeoxycholic Acid	516-50-7	Bile acids	HMDB0000896/C1160-000/CAS516-50-7
Tryptophanamide	1760501	Indoles and derivatives	HMDB0013318
3,6-Diketocholanic Acid Methyl Ester	6868-73-1	Bile acids	C1305-000
Dehydrolithocholic Acid Methyl Ester	1553-56-6	Bile acids	C1765-000
23-Nordeoxycholic Acid Diacetate	53608-86-9	Bile acids	N2020-000
23-Norcholic Acid Diacetate	60696-62-0	Bile acids	N2454-000
Pentadecanoic acid	1002-84-2	Fatty acids	HMDB0000826
Palmitelaidic acid	10030-73-6	Fatty acids	HMDB0012328
Glutarylcarnitine(C5DC)	102636-82-8	Carnitines or Acyl carnitines	HMDB0013130
Etienic Acid	10325-79-8	Bile acids	A8300-000/CAS 10325-79-8
Octyl gallate	1034-01-1	Benzenoids	HMDB0033375
2-Phenylethyl 3-phenyl-2-propenoate	103-53-7	Phenylpropanoids and polyketides	HMDB0035018
Eicosapentaenoic acid EPA	10417-94-4	Fatty acids	HMDB0001999
Urocanic acid	104-98-3	Organic acids	HMDB0000301
Lithocholenic Acid	1053-37-8	Bile acids	C2700-000/CAS 1053-37-8
Aminolevulinic acid	106-60-5	Amino acids and Peptides	HMDB0001149
O-Phosphoryl-ethanolamine	1071-23-4	Organic acids	HMDB0000224

Betaine	107-43-7	Amino acids and Peptides	HMDB0000043
(S)-Lipoic acid	1077-27-6	Organoheterocyclic	HMDB0014312
Butyric acid	107-92-6	Fatty acids	HMDB0000039
Beta-Alanine	107-95-9	Amino acids and Peptides	HMDB0000056
Sarcosine	107-97-1	Amino acids and Peptides	HMDB0000271
Valeric acid	109-52-4	Fatty acids	HMDB0000892
Fumaric acid	110-17-8	Organic acids	HMDB0000134
Glutaric acid	110-94-1	Organic acids	HMDB0000661
Pimelic acid	111-16-0	Fatty acids	HMDB0000857
Sebacic acid	111-20-6	Fatty acids	HMDB0000792
cis,cis-Muconic acid	1119-72-8	Fatty acids	HMDB0006331
Nonanoic acid	112-05-0	Fatty acids	HMDB0000847
L-Gulonolactone	1128-23-0	Organoheterocyclic	HMDB0003466
Ferulic acid	1135-24-6	Phenylpropanoids and polyketides	HMDB0000954
N-Methylnicotinamide	114-33-0	Pyridines and derivatives	HMDB0003152
Methylcysteine	1187-84-4	Amino acids and Peptides	HMDB0002108
2-Methylheptanoic Acid	1188-02-9	Fatty acids	HMDB0031587
N-Acetylleucine	1188-21-2	Amino acids and Peptides	HMDB0011756
2-Methylbenzoic acid	118-90-1	Benzenoids	HMDB0002340
L-Homocitrulline	1190-49-4	Amino acids and Peptides	HMDB0000679
Tranexamic Acid	1197-18-8	Amino acids and Peptides	HMDB0014447
3,4-Dihydro-2H-1-benzopyran-2-one	119-84-6	Phenylpropanoids and polyketides	HMDB0036626
p-Hydroxymandelic acid	1198-84-1	Benzenoids	HMDB0000822
2-HYDROXY-4-(METHYLTHIO)BUTANOATE	120-91-2	Fatty acids	HMDB0037115
Vanillic acid	121-34-6	Benzenoids	HMDB0000484

N-acetyltryptophan	1218-34-4	Amino acids and Peptides	HMDB0013713
4-Hydroxybenzaldehyde	123-08-0	Benzenoids	HMDB0011718
Levulinic acid	123-76-2	Organic acids	HMDB0000720
Azelaic acid	123-99-9	Fatty acids	HMDB0000784
Adipic acid	124-04-9	Fatty acids	HMDB0000448
METHYL-LITHOCHOLATE	1249-75-8	Bile acids	C1450-000/CAS1249-75-8
Ursodeoxycholic acid	128-13-2	Bile acids	HMDB0000946
Glycohyodeoxycholic Acid	13042-33-6	Bile acids	C0865-000/CAS 13042-33-6
2-hydroxyglutaric acid	13095-48-2	Fatty acids	HMDB0000694
Ureidosuccinic acid	13184-27-5	Organic acids	HMDB0000828
Vanillymandelic Acid	13244-77-4	Organic acids	HMDB0000291
3-Indolebutyric acid	133-32-4	Indoles and derivatives	HMDB0002096
Hydroxyisocaproic acid	13748-90-8	Fatty acids	HMDB0000746
Arabinonic acid	13752-83-5	Carbohydrates	HMDB0000539
Erythronic acid	13752-84-6	Carbohydrates	HMDB0000613
Irbesartan	138402-11-6	Benzenoids	HMDB0015163
3,4-Dihydroxybenzaldehyde	139-85-5	Benzenoids	HMDB0059965
trans-Cinnamic acid	140-10-3	Benzenoids	HMDB0000930
Ricinoleic acid	141-22-0	Fatty acids	HMDB0034297
Dodecanoic acid	143-07-7	Fatty acids	HMDB0000638
2,3-Diphosphoglyceric acid	14438-19-8	Carbohydrates	HMDB0001294
Oxalic acid	144-62-7	Organic acids	HMDB0002329
Cholic Acid Methyl Ester	1448-36-8	Bile acids	C1950-000/CAS 1448-36-8
Pregnenolone	145-13-1	Steroids and steroid derivatives	HMDB0000253
Selenomethionine	1464-42-2	Amino acids and Peptides	HMDB0003966

Xanthosine	146-80-5	Nucleosides	HMDB0000299
3-Indoleglyoxylic acid	1477-49-2	Indoles and derivatives	HMDB0242143
2-Indolecarboxylic acid	1477-50-5	Indoles and derivatives	HMDB0002285
L-Proline	147-85-3	Amino acids and Peptides	HMDB0000162
L-Alpha-aminobutyric acid	1492-24-6	Organic acids	HMDB0000452
Decanoylcarnitine (C10)	1492-27-9	Carnitines or Acyl carnitines	HMDB0000651
2-Ethylhexanoic acid	149-57-5	Fatty acids	HMDB0031230
Gallic acid	149-91-7	Benzenoids	HMDB0005807
Monomethyl glutaric acid	1501-27-5	Fatty acids	HMDB0000858
p-Aminobenzoic acid	150-13-0	Benzenoids	HMDB0001392
Bicine	150-25-4	Organic acids	HMDB0011727
3-Methyloxindole	1504-06-9	Indoles and derivatives	HMDB0304943
Etianic Acid	15173-54-3	Bile acids	A2210-000/CAS15173-54-3
7-Methylguanosine	15313-37-8	Nucleosides	HMDB0001107
2-Deoxyglucose	154-17-6	Carbohydrates	HMDB0062477
p-Hydroxyphenylacetic acid	156-38-7	Benzenoids	HMDB0000020
4-Hydroxyphenylpyruvic acid	156-39-8	Benzenoids	HMDB0000707
L-Homoarginine	156-86-5	Amino acids and Peptides	HMDB0000670
1-Methyladenosine	15763-06-1	Nucleosides	HMDB0003331
L-Thyronine	1596-67-4	Amino acids and Peptides	HMDB0000667
Isovalerylglycine	16284-60-9	Amino acids and Peptides	HMDB0000678
3-Hydroxyhippuric	1637-75-8	Benzenoids	HMDB0006116
Doxepin	1668-19-5	Organoheterocyclic	HMDB0015273
10-Hydroxydecanoic acid	1679-53-4	Organic acids	HMDB0244272
CIS-11,14,17-EICOSATRIENOIC ACID	17046-59-2	Fatty acids	HMDB0060039

Propionylcarnitine	17298-37-2	Carnitines or Acyl carnitines	HMDB0000824
13C,16C-DOCOSADIENOIC ACID	17735-98-7	Fatty acids	HMDB0061714
D-Aspartic acid	1783-96-6	Amino acids and Peptides	HMDB0006483
3-Methoxyphenylacetic acid	1798-09-0	Benzenoids	HMDB0059969
Benzenebutanoic acid	1821-12-1	Benzenoids	HMDB0000543
N-Acetylneuraminic Acid	19342-33-7	Carbohydrates	HMDB0000230
N-alpha-Acetyl-L-lysine	1946-82-3	Amino acids and Peptides	HMDB0000446
Glycylvaline	1963-21-9	Amino acids and Peptides	HMDB0028854
L-3-Phenyllactic acid	20312-36-1	Benzenoids	HMDB0000748
5-Aminoimidazole-4-carboxamide	21299-72-9	Organoheterocyclic	HMDB0003192
S-Allylcysteine	21593-77-1	Amino acids and Peptides	HMDB0034323
Methylsuccinic acid	2174-58-5	Fatty acids	HMDB0001844
Nifedipine	21829-25-4	Organoheterocyclic	HMDB0015247
5β-CHOLANIC ACID METHYL ESTER	2204-14-0	Bile acids	C0825-000/CAS2204-14-0
2-Acetylpyrazine	22047-25-2	Organooxygen	HMDB0031861
1-Aminocyclopropanecarboxylic acid	22059-21-8	Amino acids and Peptides	HMDB0036458
ISOALLOLITHOCHOLIC ACID	2276-93-9	Bile acids	C0700-000/CAS 2276-93-9
Aspartame	22839-47-0	Amino acids and Peptides	HMDB0001894
Palmitoylcarnitine	2364-67-2	Carnitines or Acyl carnitines	HMDB0000222
S-Carboxymethyl-L-cysteine	2387-59-9	Amino acids and Peptides	HMDB0029415
Beta-Muricholic Acid	2393-59-1	Bile acids	C1895-000/HMDB0000415
(7Z)-Hexadecenoic Acid	2416-19-5	Fatty acids	HMDB0002186
5Z-Dodecenoic acid	2430-94-6	Fatty acids	HMDB0000529
Tricosanoic acid	2433-96-7	Fatty acids	HMDB0001160
Tauro-omega-Muricholic Acid	2456348-84-6	Bile acids	CAS2456348-84-6

3-Methyl-2-oxovaleric acid	24809-08-3	Organic acids	HMDB0000491
Docosapentaenoic acid DPA	24880-45-3	Fatty acids	HMDB0006528
N-Acetylglutamine	2490-97-3	Amino acids and Peptides	HMDB0006029
N-Acetyl-L-histidine	2497-02-1	Amino acids and Peptides	HMDB0032055
Lauroylcarnitine	25518-54-1	Carnitines or Acyl carnitines	HMDB0002250
Glucaric acid	25525-21-7	Carbohydrates	HMDB0000663
Myristoylcarnitine	25597-07-3	Carnitines or Acyl carnitines	HMDB0005066
Stearylcarnitine(C18)	25597-09-5	Carnitines or Acyl carnitines	HMDB0000848
cis-4-Hydroxy-D-proline	2584-71-6	Amino acids and Peptides	HMDB0060460
3-Mercaptolactic acid	2614-83-7	Carbohydrates	HMDB0002127
trans-Piceid	27208-80-6	Phenylpropanoids and polyketides	HMDB0030564
Alpha-N-Phenylacetyl-L-glutamine	28047-15-6	Amino acids and Peptides	HMDB0006344
N,N-Dimethyl-L-Valine	2812-32-0	Amino acids and Peptides	PubChemCID11008044
D-Arabinose	28697-53-2	Carbohydrates	HMDB0029942
Adrenic acid	28874-58-0	Fatty acids	HMDB0002226
L-Kynurenine	2922-83-0	Amino acids and Peptides	HMDB0000684
Ursocholic Acid	2955-27-3	Bile acids	HMDB0000917
Taurohyodeoxycholic Acid	38411-85-7	Bile acids	C0890-000/CAS 38411-85-7
10Z-Heptadecenoic acid	29743-97-3	Fatty acids	HMDB0060038
Glyoxylic acid	298-12-4	Organic acids	HMDB0000119
Carbamazepine	298-46-4	Organoheterocyclic	HMDB0014704
Indoleacrylic acid	29953-71-7	Indoles and derivatives	HMDB0000734
4-Acetamidobutanoic acid	3025-96-5	Amino acids and Peptides	HMDB0060265
all-trans-Retinoic acid	302-79-4	Phenylpropanoids and polyketides	HMDB0001852
Choleic Acid	302-95-4	Bile acids	C1070-000/CAS 302-95-4

4-ANDROSTEN-3-ONE-17β-CARBOXYLIC ACID	302-97-6	Bile acids	A7100-000/CAS302-97-6
Clomipramine	303-49-1	Organoheterocyclic	HMDB0015372
L-Acetylcarnitine	3040-38-8	Carnitines or Acyl carnitines	HMDB0000201
Chenodeoxycholic Acid Methyl Ester	3057-04-3	Bile acids	C0975-000
3-Methyladipic acid	3058-01-3	Fatty acids	HMDB0000555
Carnosine	305-84-0	Amino acids and Peptides	HMDB0000033
Homovanillic acid	306-08-1	Benzenoids	HMDB0000118
Alanylphenylalanine	3061-90-3	Amino acids and Peptides	HMDB0028694
Hydroxyphenyllactic acid	306-23-0	Fatty acids	HMDB0000755
Isovelarylcarnitine	31023-24-2	Carnitines or Acyl carnitines	HMDB0000688
L-Pipecolic acid	3105-95-1	Amino acids and Peptides	HMDB0000716
Isocitric acid	320-77-4	Organic acids	HMDB0000193
β-(m-Hydroxyphenyl)hydracrylic Acid	3247-75-4	Phenylpropanoids and polyketides	HMDB0002643
(S)C(S)S-S-Methylcysteine sulfoxide	32726-14-0	Amino acids and Peptides	HMDB0029432
L-Norleucine	327-57-1	Amino acids and Peptides	HMDB0001645
Oxalacetic acid	328-42-7	Organic acids	HMDB0000223
Oxoglutaric acid	328-50-7	Organic acids	HMDB0000208
Caffeic acid	331-39-5	Phenylpropanoids and polyketides	HMDB0001964
1-Methylhistidine	332-80-9	Amino acids and Peptides	HMDB0000001
Decanoic acid	334-48-5	Fatty acids	HMDB0000511
2-Hydroxybutyric acid	3347-90-8	Fatty acids	HMDB0000008
Glycodehydrocholic Acid	3415-45-0	Bile acids	C2020-000/CAS 3415-45-0
Glucosamine	3416-24-8	Benzenoids	HMDB0001514
4-ANDROSTEN-11β, 17α-DIOL-3-ONE-17β-CARBOXYLIC ACID	3597-45-3	Bile acids	A5740-000/CAS3597-45-3

12-hydroxystearic acid	36377-33-0	Fatty acids	HMDB0061706
Linoleyl carnitine	36816-10-1	Carnitines or Acyl carnitines	HMDB0006469
But-2-enoic acid	3724-65-0	Fatty acids	HMDB0010720
Citrulline	372-75-8	Amino acids and Peptides	HMDB0000904
Palmitoleic acid	373-49-9	Fatty acids	HMDB0003229
2-Hydroxy-2-methylbutyric acid	3739-30-8	Amino acids and Peptides	HMDB0001987
Betamethasone	378-44-9	Steroids and steroid derivatives	HMDB0014586
Oleoylcarnitine	38677-66-6	Carnitines or Acyl carnitines	HMDB0005065
5β-ANDROSTAN-3α-OL-17β-CARBOXYLIC ACID	38775-99-4	Bile acids	A3370-000/CAS38775-99-4
Indolepyruvate	392-12-1	Indoles and derivatives	HMDB0060484
Alanylglutamine	39537-23-0	Amino acids and Peptides	HMDB0028685
2,3-Diaminopropionic acid	4033-39-0	Amino acids and Peptides	HMDB0002006
Lithocholic Acid Acetate	4057-84-5	Bile acids	C1421-000
Phosphoserine	407-41-0	Amino acids and Peptides	HMDB0000272
N-(4-Aminobenzoyl)-L-glutamic Acid	4271-30-1	Amino acids and Peptides	CAS4271-30-1
Lithocholic acid	434-13-9	Bile acids	HMDB0000761
5-Hydroxy-L-tryptophan	4350-09-8	Indoles and derivatives	HMDB0000472
6-Methyladenine	443-72-1	Organoheterocyclic	HMDB0002099
Homogentisic acid	451-13-8	Benzenoids	HMDB0000130
Curcumin	458-37-7	Phenylpropanoids and polyketides	HMDB0002269
3-Phenylbutyric acid	4593-90-2	Phenylpropanoids and polyketides	HMDB0001955
Lactulose	4618-18-2	Carbohydrates	HMDB0000740
4-Guanidinobutyric acid	463-00-3	Organic acids	HMDB0003464
Alpha-Linolenic acid	463-40-1	Fatty acids	HMDB0001388
7-Ketolithocholic Acid	4651-67-6	Bile acids	C1600-000/HMDB0000467

Glycocholic acid	475-31-0	Bile acids	HMDB0000138
Naringenin	480-41-1	Phenylpropanoids and polyketides	HMDB0002670
Santonin	481-06-1	Prenol Lipids	PubChemCID221071
3,3-Dimethylglutaric acid	4839-46-7	Fatty acids	HMDB0002441
Salicyluric acid	487-54-7	Benzenoids	HMDB0000840
Indole-3-carboxaldehyde	487-89-8	Indoles and derivatives	HMDB0029737
Ribitol	488-81-3	Carbohydrates	HMDB0000508
Epicatechin	490-46-0	Phenylpropanoids and polyketides	HMDB0001871
Gentisic acid	490-79-9	Benzenoids	HMDB0000152
Dehydroascorbic acid	490-83-5	Organoheterocyclic	HMDB0001264
Kynurenic acid	492-27-3	Quinolines and derivatives	HMDB0000715
2-Phenylpropionic acid	492-37-5	Phenylpropanoids and polyketides	HMDB0011743
Hippuric acid	495-69-2	Benzenoids	HMDB0000714
Acetovanillone	498-02-2	Organooxygen	HMDB0247918
Citraconic acid	498-23-7	Fatty acids	HMDB0000634
L-Cysteic acid	498-40-8	Amino acids and Peptides	HMDB0002757
Isomaltose	499-40-1	Carbohydrates	HMDB0002923
4-Hydroxy-3-methylbenzoic acid	499-76-3	Benzenoids	HMDB0004815
2,6-Pyridinedicarboxylic acid	499-83-2	Pyridines and derivatives	HMDB0033161
Resveratrol	501-36-0	Phenylpropanoids and polyketides	HMDB0003747
Hydrocinnamic acid	501-52-0	Phenylpropanoids and polyketides	HMDB0000764
3-Pyridylacetic acid	501-81-5	Pyridines and derivatives	HMDB0001538
Desaminotyrosine	501-97-3	Phenylpropanoids and polyketides	HMDB0002199
4-Hydroxycinnamic acid	501-98-4	Phenylpropanoids and polyketides	HMDB0002035
Hydroretrocortine	50-24-8	Steroids and steroid derivatives	HMDB0014998

Estriol	50-27-1	Steroids and steroid derivatives	HMDB0000153
Myristelaidic acid	50286-30-1	Fatty acids	HMDB0062248
Thioridazine	50-52-2	Organoheterocyclic	HMDB0014817
Suberic acid	505-48-6	Fatty acids	HMDB0000893
CIS-11-OCTADECENOIC ACID	506-17-2	Fatty acids	HMDB0240219
Linolelaidic acid	506-21-8	Fatty acids	HMDB0006270
Arachidonic acid	506-32-1	Fatty acids	HMDB0001043
D-Glucose	50-99-7	Carbohydrates	HMDB0000122
12-Ketolithocholic acid	5130-29-0	Bile acids	C1650-000/HMDB0000328
4-Hydroxyproline	51-35-4	Amino acids and Peptides	HMDB0000725
Etienic Acid Acetate	51424-66-9	Bile acids	A8301-000
O-Acetylserine	5147-00-2	Amino acids and Peptides	HMDB0003011
Cimetidine	51481-61-9	Organoheterocyclic	HMDB0014644
Retinal	514-85-2	Retinoids	HMDB0001358
Taurochenodeoxycholic Acid	516-35-8	Bile acids	C0990-000/HMDB0000951
Dioxolithocholic Acid	517-33-9	Bile acids	CAS517-33-9
Diosmetin	520-34-3	Phenylpropanoids and polyketides	HMDB0029676
Medroxyprogesterone	520-85-4	Steroids and steroid derivatives	HMDB0001939
Gluconic acid	526-95-4	Carbohydrates	HMDB0000625
Galactaric acid	526-99-8	Carbohydrates	HMDB0000639
Syringic acid	530-57-4	Benzenoids	HMDB0002085
Sinapic acid	530-59-6	Phenylpropanoids and polyketides	HMDB0032616
Cortisone	53-06-5	Steroids and steroid derivatives	HMDB0002802
Nordeoxycholic Acid	53608-86-9	Bile acids	N2000-000/CAS 53608-86-9
Acetoacetic acid	541-50-4	Organic acids	HMDB0000060

Acetylglycine	543-24-8	Amino acids and Peptides	HMDB0000532
Idoxuridine	54-42-2	Nucleosides	HMDB0014394
Myristic acid	544-63-8	Fatty acids	HMDB0000806
Myristoleic acid	544-64-9	Fatty acids	HMDB0002000
Hyocholic acid	547-75-1	Bile acids	C1850-000/HMDB0000760
3-Hydroxyanthranilic acid	548-93-6	Benzenoids	HMDB0001476
D-Xylulose	551-84-8	Carbohydrates	HMDB0001644
Acesulfame	55589-62-3	Organic acids	HMDB0033585
Glycyl-glycine	556-50-3	Amino acids and Peptides	HMDB0011733
11C,14C-EICOSADIENOIC ACID	5598-38-9	Fatty acids	HMDB0005060
Gamma-Aminobutyric acid	56-12-2	Amino acids and Peptides	HMDB0000112
Glycine	56-40-6	Amino acids and Peptides	HMDB0000123
L-Alanine	56-41-7	Amino acids and Peptides	HMDB0000161
L-Serine	56-45-1	Amino acids and Peptides	HMDB0000187
isodeoxycholic acid	566-17-6	Bile acids	C1170-000/HMDB0002536
3β-HYDROXY-BISNOR-5-CHOLENIC ACID	566-77-8	Bile acids	B0700-000/CAS566-77-8
Glyceraldehyde	56-82-6	Carbohydrates	HMDB0001051
L-Aspartic acid	56-84-8	Amino acids and Peptides	HMDB0000191
L-Glutamine	56-85-9	Amino acids and Peptides	HMDB0000641
L-Glutamic acid	56-86-0	Amino acids and Peptides	HMDB0000148
L-Lysine	56-87-1	Amino acids and Peptides	HMDB0000182
3-Aminosalicylic acid	570-23-0	Benzenoids	HMDB0001972
Beta-Hyodeoxycholic Acid	570-84-3	Bile acids	HMDB0000664
Palmitic acid	57-10-3	Fatty acids	HMDB0000220
Progesterone	57-83-0	Steroids and steroid derivatives	HMDB0001830

Menadione	58-27-5	Benzenoids	HMDB0001892
Anserine	584-85-0	Amino acids and Peptides	HMDB0000194
Cis-and trans-Aconitic acid	585-84-2	Organic acids	HMDB0000072
Melibiose	585-99-9	Carbohydrates	HMDB0000048
Adenosine	58-61-7	Nucleosides	HMDB0000050
Inosine	58-63-9	Nucleosides	HMDB0000195
m-Coumaric acid	588-30-7	Benzenoids	HMDB0001713
D-Xylose	58-86-6	Carbohydrates	HMDB0000098
Uridine 5'-monophosphate	58-97-9	Nucleosides	HMDB0000288
Xanthurenic acid	59-00-7	Quinone and hydroquinone lipids	HMDB0000881
alpha-Tocopherol	59-02-9	Quinone and hydroquinone lipids	HMDB0001893
12-Dehydrocholic Acid Diacetate	59274-67-8	Bile acids	C1220-000
Folic acid	59-30-3	Benzenoids	HMDB0000121
alpha-Hydroxyisobutyric acid	594-61-6	Organic acids	HMDB0000729
Nicotinic acid	59-67-6	Pyridines and derivatives	HMDB0001488
2,2-Dimethylsuccinic acid	597-43-3	Fatty acids	HMDB0002074
Citramalic acid	597-44-4	Fatty acids	HMDB0000426
Edetic Acid	60-00-4	Organic acids	HMDB0015109
12-Tridecanoic acid	6006-06-0	Bile acids	CAS6006-06-0
2-Methylcitric acid	6061-96-7	Organic acids	HMDB0000379
23-Norcholic Acid	60696-62-0	Bile acids	N2450-000/CAS 60696-62-0
Cyclic AMP	60-92-4	Nucleosides	HMDB0000058
Phenylglyoxylic acid	611-73-4	Benzenoids	HMDB0001587
Adenosine monophosphate	61-19-8	Nucleosides	HMDB0000045
2-Hydroxycinnamic acid	614-60-8	Fatty acids	HMDB0002641

Ortho-Hydroxyphenylacetic acid	614-75-5	Benzenoids	HMDB0000669
Hydroxyoctanoic acid	617-73-2	Fatty acids	HMDB0000711
4-Aminohippuric acid	61-78-9	Benzenoids	HMDB0001867
Paroxetine	61869-08-7	Organoheterocyclic	HMDB0014853
L-Leucine	61-90-5	Amino acids and Peptides	HMDB0000687
3-Hydroxyphenylacetic acid	621-37-4	Benzenoids	HMDB0000440
3-Nitrotyrosine	621-44-3	Amino acids and Peptides	HMDB0001904
Glycerol trihexanoate	621-70-5	Glycerolipids	HMDB0031125
Docosahexaenoic acid DHA	6217-54-5	Fatty acids	HMDB0002183
Cinnamic acid	621-82-9	Phenylpropanoids and polyketides	HMDB0000567
3-Hydroxyisovaleric acid	625-08-1	Fatty acids	HMDB0000754
3-Hydroxybutyric acid	625-71-8	Organic acids	HMDB0000011
2-Aminoisobutyric acid	62-57-7	Amino acids and Peptides	HMDB0001906
Methylglutaric acid	626-51-7	Fatty acids	HMDB0000752
L-homocystine	626-72-2	Amino acids and Peptides	HMDB0000676
4-ANDROSTEN-17α-OL-3-ONE-17β-CARBOXYLIC ACID	633-29-4	Bile acids	A6905-000/CAS633-29-4
5-Hydroxymethyl-2-furancarboxylic acid	6338-41-6	Organoheterocyclic	HMDB0002432
L-Methionine	63-68-3	Amino acids and Peptides	HMDB0000696
N-Methyl-D-aspartic acid	6384-92-5	Amino acids and Peptides	HMDB0002393
L-Phenylalanine	63-91-2	Amino acids and Peptides	HMDB0000159
Traumatic acid	6402-36-4	Fatty acids	HMDB0000933
Glycochenodeoxycholic acid	640-79-9	Bile acids	HMDB0000637
Apocholic Acid	641-81-6	Bile acids	C2500-000/CAS 641-81-6
Acetic acid	64-19-7	Fatty acids	HMDB0000042

Ribonic acid	642-98-8	Carbohydrates	HMDB0000867
Glycoursodeoxycholic acid	64480-66-6	Bile acids	HMDB0000708
11-Dodecenoic acid	65423-25-8	Fatty acids	HMDB0032248
Cytidine triphosphate	65-47-4	Nucleosides	HMDB0000082
Benzoic acid	65-85-0	Benzenoids	HMDB0001870
Orotic acid	65-86-1	Organoheterocyclic	HMDB0000226
5-Aminopentanoic acid	660-88-8	Amino acids and Peptides	HMDB0003355
Murocholic acid	668-49-5	Bile acids	C0910-000/HMDB0000811
thiamine disulfide	67-16-3	Organoheterocyclic	PubChemCID3001376
L-Homoserine	672-15-1	Amino acids and Peptides	HMDB0000719
3,6-Diketocholanic Acid	6868-73-1	Bile acids	C1280-000/CAS 006868-73-1
9(11), (5β)-CHOLENIC ACID-3α-OL-12-ONE	6874-18-6	Bile acids	C2670-000/CAS6874-18-6
3-Oxoadipic acid	689-31-6	Organic acids and derivatives	HMDB0000398
N-Acetylproline	68-95-1	Amino acids and Peptides	HMDB0094701
Dodecanedioic acid	693-23-2	Fatty acids	HMDB0000623
L-Histidine	71-00-1	Amino acids and Peptides	HMDB0000177
Meloxicam	71125-38-7	Organoheterocyclic	HMDB0014952
L-Valine	72-18-4	Amino acids and Peptides	HMDB0000883
L-threonine	72-19-5	Amino acids and Peptides	HMDB0000167
Catechin	7295-85-4	Phenylpropanoids and polyketides	HMDB0002780
Threonic acid	7306-96-9	Organic acids	HMDB0000943
8,11,14-Eicosatrienoic acid	7324-41-6	Fatty acids	HMDB0002925
Melatonin	73-31-4	Indoles and derivatives	HMDB0001389
L-Isoleucine	73-32-5	Amino acids and Peptides	HMDB0000172
N-Phenylacetylphenylalanine	738-75-0	Amino acids and Peptides	HMDB0002372

L-Arginine	74-79-3	Amino acids and Peptides	HMDB0000517
N-Acetyl-D-glucosamine	7512-17-6	Carbohydrates	HMDB0000215
Alpha-ketoisovaleric acid	759-05-7	Organic acids	HMDB0000019
Diethyl oxalpropionate	759-65-9	Organic acids	HMDB0032306
Indole-3-carboxylic acid	771-50-6	Indoles and derivatives	HMDB0003320
3,4-Dihydroxymandelic acid	775-01-9	Benzenoids	HMDB0001866
3,7-Diketocholanic Acid Methyl Ester	7753-72-2	Bile acids	C1355-000/CAS 7753-72-2
Piperine	7780-20-3	Alkaloids and derivatives	HMDB0029377
Citric acid	77-92-9	Organic acids	HMDB0000094
Beta-Ursodeoxycholic Acid	78919-26-3	Bile acids	HMDB0000686
Propanoic acid	79-09-4	Fatty acids	HMDB0000237
Glycolic acid	79-14-1	Organic acids	HMDB0000115
Pantolactone	79-50-5	Organoheterocyclic	HMDB0059876
Dehydrocholic Acid	81-23-2	Bile acids	C2000-000/CAS 81-23-2
Cholic Acid	81-25-4	Bile acids	C1900-000/HMDB0000619
Ketoleucine	816-66-0	Organic acids	HMDB0000695
Tetradecanedioic acid	821-38-5	Fatty acids	HMDB0000872
Phenyllactic acid	828-01-3	Benzenoids	HMDB0000779
3-Indolepropionic acid	830-96-6	Indoles and derivatives	HMDB0002302
Deoxycholic acid	83-44-3	Bile acids	HMDB0000626
Hyodeoxycholic acid	83-49-8	Bile acids	HMDB0000733
Pyridoxal 5'-phosphate	853645-22-4	Benzenoids	HMDB0001491
3,7-Diketocholanic Acid	859-97-2	Bile acids	C1330-000/CAS 859-97-2
Glycylleucine	869-19-2	Amino acids and Peptides	HMDB0000759
Indoleacetic acid	87-51-4	Organic acids	HMDB0000197

Mannitol	87-78-5	Carbohydrates	HMDB0000765
L-Sorbose	87-79-6	Carbohydrates	HMDB0001266
myo-Inositol	87-89-8	Organooxygen	HMDB0000211
Diethyl tartrate	87-91-2	Organic acids	HMDB0033584
5β-CHOLANIC ACID-3α, 6α-DIOL-7-ONE	88725-42-2	Bile acids	C1198-000/CAS88725-42-2
Phthalic acid	88-99-3	Benzenoids	HMDB0002107
Quinolinic acid	89-00-9	Organic acids	HMDB0000232
3,4,5-Trimethoxycinnamic acid	90-50-6	Phenylpropanoids and polyketides	HMDB0002511
Mandelic acid	90-64-2	Benzenoids	HMDB0000703
D-Gluconolactone	90-80-2	Carbohydrates	HMDB0000150
Malonylcarnitine	910825-21-7	Carnitines or Acyl carnitines	HMDB0002095
7-Dehydrocholic acid	911-40-0	Bile acids	C1250-000/HMDB0000391
Homoveratric acid	93-40-3	Benzenoids	HMDB0000434
4-Hydroxyphenyl-2-propionic acid	938-96-5	Phenylpropanoids and polyketides	HMDB0041683
Allantoin	97-59-6	Imidazoles	HMDB0000462
2-Methylvaleric acid	97-61-0	Fatty acids	HMDB0031580
Itaconic acid	97-65-4	Organic acids	HMDB0002092
N-Acetyl-L-alanine	97-69-8	Amino acids and Peptides	HMDB0000766
s-(5'-Adenosyl)-L-homocysteine	979-92-0	Nucleosides	HMDB0000939
1,(5α)-ANDROSTEN-4-AZA-3-ONE-17β-CARBOXYLIC ACID	98319-26-7	Bile acids	A4355-000/CAS98319-26-7
Cyclohexanecarboxylic Acid	98-89-5	Organic acids	HMDB0031342
Epigallocatechin gallate	989-51-5	Phenylpropanoids and polyketides	HMDB0003153
Picolinic acid	98-98-6	Pyridines and derivatives	HMDB0002243
m-Aminobenzoic acid	99-05-8	Benzenoids	HMDB0001891

3,5-Dihydroxybenzoic acid	99-10-5	Benzenoids	HMDB0013677
1,2,3-Propanetricarboxylic acid	99-14-9	Organic acids	HMDB0031193
Protocatechuic acid	99-50-3	Benzenoids	HMDB0001856
Valproic acid	99-66-1	Fatty acids	HMDB0001877
N-Acetyl-L-aspartic acid	997-55-7	Amino acids and Peptides	HMDB0000812
4-Hydroxybenzoic acid	99-96-7	Benzenoids	HMDB0000500
Etiadienic Acid	#N/A	Bile acids	A0680-000
Etiadienic Acid 3-Acetate	#N/A	Bile acids	A0681-000
Etiadienic Acid Methyl Ester	#N/A	Bile acids	A0689-000
5β-CHOLANIC ACID-12α-BROMO-3, 11-DIONE METHYL ESTER	#N/A	Bile acids	C0804-000
7-Ketolithocholic Acid Acetate Methyl Ester	#N/A	Bile acids	C1610-000
5-CHOLENIC ACID-3β-OL METHYL ESTER, 3-METHYL ETHER	#N/A	Bile acids	C2471-000/CAS20231-57-6
9(11), (5β)-CHOLENIC ACID-3α-OL-12-ONE ACETATE METHYL ESTER	#N/A	Bile acids	C2685-000
Leucyl-Glycine	#N/A	Amino acids and Peptides	HMDB0028929
N-Methyl-L-glutamic acid	#N/A	Amino acids and Peptides	HMDB0062660
N-carbamoylglutamic Acid	#N/A	Amino acids and Peptides	HMDB0062795
23-Nordeoxycholic Acid Methyl Ester	#N/A	Bile acids	N2070-000
DEOXYCHENOCHOLIC ACID	#N/A	Bile acids	HM4000111
5β-CHOLANIC ACID-3α-OLN-(CARBOXYMETHYL)	#N/A	Bile acids	HM4000113
5β-ANDROSTAN-3-ONE-17β-CARBOXYLIC ACID METHYL ESTER	#N/A	Bile acids	HM4000108

5β-CHOLANIC ACID-3α, 12α-DIOL N-(2-SULPHOETHYL)-AMIDE	#N/A	Bile acids	HM4000109
